# A Bayesian Approach to the Overlap Analysis of Epidemiologically Linked Traits

**DOI:** 10.1002/gepi.21919

**Published:** 2015-09-28

**Authors:** Jennifer L. Asimit, Kalliope Panoutsopoulou, Eleanor Wheeler, Sonja I. Berndt, the GIANT consortium, the arcOGEN consortium, Heather J. Cordell, Andrew P. Morris, Eleftheria Zeggini, Inês Barroso

**Affiliations:** ^1^ Wellcome Trust Sanger InstituteHinxtonCambridgeUnited Kingdom; ^2^ Division of Cancer Epidemiology and GeneticsNational Cancer InstituteUS National Institutes of HealthBethesdaMarylandUnited States of America; ^3^ Institute of Genetic MedicineNewcastle UniversityNewcastle upon TyneUnited Kingdom; ^4^ Wellcome Trust Centre for Human GeneticsUniversity of OxfordOxfordUnited Kingdom; ^5^ Department of BiostatisticsUniversity of LiverpoolLiverpoolUnited Kingdom

**Keywords:** Bayes’ factor, *P*‐value, obesity, osteoarthritis, overlap analysis, threshold calibration

## Abstract

Diseases often cooccur in individuals more often than expected by chance, and may be explained by shared underlying genetic etiology. A common approach to genetic overlap analyses is to use summary genome‐wide association study data to identify single‐nucleotide polymorphisms (SNPs) that are associated with multiple traits at a selected *P*‐value threshold. However, *P*‐values do not account for differences in power, whereas Bayes’ factors (*BFs*) do, and may be approximated using summary statistics. We use simulation studies to compare the power of frequentist and Bayesian approaches with overlap analyses, and to decide on appropriate thresholds for comparison between the two methods. It is empirically illustrated that *BFs* have the advantage over *P*‐values of a decreasing type I error rate as study size increases for single‐disease associations. Consequently, the overlap analysis of traits from different‐sized studies encounters issues in fair *P*‐value threshold selection, whereas *BFs* are adjusted automatically. Extensive simulations show that Bayesian overlap analyses tend to have higher power than those that assess association strength with *P*‐values, particularly in low‐power scenarios. Calibration tables between *BFs* and *P*‐values are provided for a range of sample sizes, as well as an approximation approach for sample sizes that are not in the calibration table. Although *P*‐values are sometimes thought more intuitive, these tables assist in removing the opaqueness of Bayesian thresholds and may also be used in the selection of a *BF* threshold to meet a certain type I error rate. An application of our methods is used to identify variants associated with both obesity and osteoarthritis.

## Introduction

Multiple health disorders may afflict an individual at any given time, and several such disorders frequently cooccur more often than expected by chance. In contrast, certain pairs of disorders are rarely observed in the same individual, such that the presence of one disease appears to reduce the risk of developing the other. The cooccurrence of complex disorders with a genetic component significantly more, or significantly less, frequently than expected by chance suggests that there might be shared genetic variants that predispose to multiple disorders, or that protect against some disorders while predisposing to others. For instance, there is an increased osteoarthritis (OA) risk of 1.4–1.9 in the obesity class (body mass index (*BMI*) > 28 kg/m^2^) [Wilkin and Voss, 2005], and a genetic overlap between OA and obesity has been identified and replicated at *FTO* [Elliott et al., 2012; Panoutsopoulou et al., 2013]. In another example, individuals with schizophrenia have a fourfold higher prevalence of type 2 diabetes (20%), compared with the general population. Though some of this increased diabetes risk could be due to drug effects [Lin and Shuldiner, 2010; Salviato Balbão et al., 2014], there is also evidence of shared genetic etiology [Lin and Shuldiner, 2010].

### Current Approaches and Limitations

Often summary statistics, rather than raw data, are available when data are shared from multiple studies, and association is often assessed by *P*‐value. In planned genetic overlap analyses of two traits, there are a few approaches that have been put to use to identify variants and/or genes associated with both traits. One method is to check if any of the associated variants for one trait are associated with the other trait or fall within its candidate genes. For example, in a genome‐wide association study (GWAS) of Crohn's disease, associated single‐nucleotide polymorphisms (SNPs) were identified in the same intron of *CDKAL1* that harbors SNPs associated with type 2 diabetes, and it was shown that the associated alleles for the two diseases are not correlated [Barrett et al., 2008].

Alternatively, the results from the marginal GWAS of each trait may be analyzed in parallel to identify overlapping associated variants based on a *P*‐value significance threshold selected for both studies. In order to test whether the number of significant variants for both traits is more than expected by chance, approximate independence among the SNPs is required so that contingency table methods may be applied. A set of SNPs with low linkage disequilibrium (LD) can be formed by LD pruning. However, when deciding between one of two SNPs in LD to remove, it is usually preferred to retain the SNP with stronger evidence of association with a trait. As there are two traits, this is complicated by the restriction that the same set of pruned SNPs is required for both traits. That is, only one measure of association strength may be considered when deciding the removal of one of two SNPs in LD.

In an overlap analysis of osteoarthritis with *BMI* and height, SNPs were pruned based on the association metrics of the trait with the larger sample size [Elliott et al., 2012]. A caveat of this approach is the lack of symmetry, because the pruned set of SNPs will differ depending on the trait selected for pruning. A contingency table comparing the number of significant/nonsignificant variants against trait 1/trait 2 was then used to test for an excess of signals for both traits [Elliott et al., 2012]. However, this approach tests for an enrichment of signals for the two traits and considers the information at each SNP independently between the two traits without simultaneously taking into account the SNP association information for both traits; that is, the fact that the data for traits 1 and 2 occur as a pair at each SNP.

Overlapping loci between schizophrenia and bipolar disorder, between prostate cancer and cardiovascular disease risk factors (e.g., blood lipids), as well as between systolic blood pressure and each of several associated phenotypes, were identified by testing individual SNPs using GWAS summary statistics and a genetic pleiotropy‐informed conditional false discovery rate (FDR) method and conjunction FDR [Andreassen et al., 2013, 2014a,b]. Both the conditional FDR and conjunction FDR are in a Bayesian framework, but rely on probabilities that arise from comparisons of marginal *P*‐values for the two traits at a given SNP.

A subset‐based approach was proposed for the meta‐analysis of related but distinct traits and has been applied to identify shared risk loci among different cancer types [Bhattacharjee et al., 2012; Wang et al., 2014]. This method evaluated evidence of association at an SNP for any given subset of the studies by combining their weighted test statistics. The approach allows for heterogeneity among the studies in that some studies may have no effect, and is also applicable to heterogeneous disease subtypes. However, this method is more advantageous for more than two studies or traits. For two studies or traits, the primary set of interest is the full set of two studies rather than a subset of one of the two, and the test statistic for the full set is essentially that from a pooled analysis of the studies.

When *P*‐values are used to assess variants for association with two traits (each coming from a different study), any power differences between the two studies are not accounted for. In particular, *P*‐values are influenced by the same factors that affect power—namely, sample size and minor allele frequency (*MAF*). Although for a fixed *P*‐value threshold power to detect a disease‐associated variant increases with sample size, the type I error rate remains the same as the *P*‐value threshold, irrespective of sample size.

Rather than focusing on *P*‐values, a Bayesian approach may be employed, which takes into account the power of the study through the incorporation of the variance of the effect estimate *V* in the calculation of the approximate Bayes’ factor (*ABF*; discussed further in next section) [Wakefield, 2009]. In contrast to the *P*‐value, the *ABF* depends on both the usual Wald statistic ($z^{2}={\hat{\beta }}^{2}/V$) and V, whereas the *P*‐value depends only on the Wald statistic. Therefore, because power is affected by sample size, the *ABFs* from different study sizes are comparable, whereas *P*‐values do not account for the differing powers of the tests. Bayesian approaches to analysis are sometimes considered less appealing than *P*‐values due to their higher level of complexity, but the advantage of *ABFs* being directly comparable across studies may be crucial when studies of different powers are to be jointly analyzed.

To assist in performing comparisons between the frequentist and Bayesian approaches, we have generated a reference table of equivalent thresholds between the two approaches for a range of sample sizes and parameter settings, which acts as a point of reference between *P*‐values and *ABFs*. This calibration table was necessary in our comparisons of the frequentist and Bayesian approaches for detecting variants associated in two traits, and may also be of more general use when comparing frequentist and Bayesian versions of a method. In addition, the calibration table removes some of the opaqueness of Bayesian thresholds by providing the false‐positive rate for a given Bayesian threshold or may assist in deciding on an *ABF* threshold to satisfy a certain type I error.

Our primary interest is in the overlap analysis of traits from two different GWAS, of differing sample size and power, as such scenarios are most likely to benefit from an *ABF* approach. We propose a method of overlap analysis when only summary statistic data are available for both traits and, in an extensive simulation study, compare the frequentist and Bayesian approaches to testing for association at a single SNP. In addition to identifying SNPs that have evidence of association in both traits, we test for an excess of overlapping associated SNPs beyond that expected by chance. The proposed methods are applied to the overlap analysis of obesity (Genetic Investigation of ANthropometric Traits (GIANT) Consortium; Berndt et al. [2013]) and knee and/or hip osteoarthritis (Arthritis Research UK Osteoarthritis Genetics (arcOGEN) Consortium; arcOGEN Consortium et al. [2012]).

## Materials and Methods

In the identification of overlapping SNPs, no assumptions of independence are needed at the SNP or sample level but more restrictive assumptions may be needed when testing for an excess of overlapping signals. In testing for more overlap than expected by chance, we assume that the traits have not been measured on the same individuals, which is likely to hold, because two different studies are of interest. Although we assume independence between the individuals, such that there is not any overlap between the control sets, we found little difference in the results when there was a shared cohort within the controls of our data application.

### BF and an Approximation

In the case‐control setting, each SNP is often tested for association with the trait by fitting a logistic regression to model the probability of disease for an individual as a function of the coded genotype *x*_j_, according to a genetic model. For example, in a strict additive model *x*_j_ = 0,1,2 minor alleles are possessed by the individual at the SNP. Letting *β* denote the effect estimate at a particular SNP, such that the odds ratio *OR* = exp(*β*), the null hypothesis of no effect (*H*_0_: *β* = 0) is compared with the alternative *H*_1_: *β*≠0. The *BF* compares how likely the observed data are under the two models and is defined by

$$BF=\frac{\mathrm{Pr}\left(data|H_{1}\right)}{\mathrm{Pr}\left(data|H_{0}\right)},$$

such that larger *BF* values indicate more evidence in favor of *H*_1_ over *H*_0_; if the data are equally probable under both hypotheses then *BF* = 1 [Stephens and Balding, 2009].

Calculation of Pr(data|*H*_1_) requires specification of a prior distribution for β under *H*_1_; this prior distribution reflects the plausibility of the various effect values before observance of the data. The probability under *H*_1_ may then be calculated by integrating over all possible values of *β*, weighted according to the prior distribution. A Normal distribution with mean 0 and variance W is often chosen as the prior distribution for the effect *β* [Stephens and Balding, 2009]. Software packages such as SNPTEST [Marchini et al., 2007] and BIMBAM [Servin and Stephens, 2007] are able to compute such *BFs* with ease.

If a logistic regression model is fit to the data, then the summary genetic association data may be used to obtain *ABFs*, regardless of availability of the phenotype and genotype data. This approximation generally aligns with the calculations output from SNPTEST and BIMBAM and has been shown to be accurate in simulated case‐control data with as little as 250 each of cases and controls [Wakefield, 2007].

Based on summary genetic association data from a regression (estimates of $\hat{\beta }\;=\log\left(\hat{O}R\right)$, and $V=\mathrm{Var}(\hat{\beta })$), for each trait an *ABF* may be calculated at each variant:

$$ABF=\sqrt{\frac{V}{V+W}}\exp\left\{\frac{W}{V+W}\frac{Z^{2}}{2}\right\},$$

where $Z=\hat{\beta \;}/\sqrt{V},\;\hat{\beta }∼N\left(\beta ,V\right),\;\beta ∼N\left(0,W\right)$ and *N(μ,σ^2^)* denotes that the random variable follows a Normal distribution with mean *μ* and variance *σ^2^* [Wakefield, 2007, 2009]. In this formulation, *W*, the prior variance of *β*, is the only parameter that requires specification. Various possibilities for *W* have been proposed in the logistic regression framework of case‐control studies, and a simple choice is for *W* to be a constant value at each variant [Wakefield, 2009]. This constant value is determined based on selection of an upper value *OR*_U_ such that with low probability *OR* > *OR*_U_. A widely used default value for the prior variance of the log‐*OR* in an additive model is *W* = 0.2^2^ [Marchini et al., 2007], which may be derived based on the assumption that with two‐sided prior probability of 0.05, *OR* > 1.48. In contrast to *P*‐values, large values of *ABF* are evidence against the null hypothesis of no trait association at the variant.

### Threshold Selection

The null hypothesis of no association at an SNP is rejected if *ABF* > *PO*/*R*, where *PO* = *π*_0_/(1 – *π*_0_) is the prior odds of no association, *π*_0_ is the prior probability that there is no association at the SNP, and *R* = type II error cost/type I error cost. The roles of *π*_0_ and *R* differ, as π_0_ influences the number of significant associations, whereas *R* determines the expected number of false discoveries and missed signals [Wakefield, 2007]. In GWAS, a Bayesian threshold is based on *R* = 1 and 1 – *π*_0_ (the prior probability of an association existing) set to 10^−4^ – 10^−6^, so that a genome‐wide threshold for log_10_ *BF* is between 4 and 6 [The Wellcome Trust Case Control Consortium, 2007].

Values of *R* greater than 1 indicate that one is in “discovery mode,” and the cost of failing to identify an associated variant is higher than the cost of falsely detecting a null associated variant. For instance, under *R* = 4, the cost of missing a true signal is four times the cost of misidentifying a null variant as associated. Therefore, when the objective is to obtain a list of candidates for followup, rather than a definitive list of signals, larger values of *R* are favored.

In overlap analyses, a less‐stringent threshold may be considered, rather than requiring genome‐wide significance to be attained at a single variant for both traits. This favors a discovery setting for detecting associations in both traits, which can subsequently be validated in further replication studies. In particular, the focus is on identifying new putative signals for downstream validation, such that more false positives are preferred over more false negatives. For example, in the overlap analysis of osteoarthritis with *BMI* and height, various *P*‐value thresholds were examined, with a focus on *α* = 10^−3^ [Elliott et al., 2012]. Likewise, we focus on *π*_0_ values of 0.99 and 0.999 to reflect that we are not searching for SNPs that are genome‐wide significant in both traits and values of *R* > 1 such that we are in “discovery mode”; genome‐wide significance would require setting *π*_0_ between 0.9999 and 0.999999 [The Wellcome Trust Case Control Consortium, 2007].

### Bayesian Approach to Overlap Analysis

Although the proposed analysis may be extended to more than two traits, for ease of exposition we focus on two traits. For each SNP at which there are summary statistic data available for both traits, the *ABF* is calculated with respect to each trait and then tested for association upon selection of *π*_0_ and *R*. Approximate independence is needed among the SNPs in order to rely on contingency table methods for analysis of the distribution of SNPs with high/low *ABF* (*ABF* above or below *PO*/*R*) over the two traits.

In the pruning of the SNPs according to both traits 1 and 2, we create new association statistics ABF* and P* that reflect the strength of evidence for association in both traits. At a given SNP, let *ABF*_1_ and *ABF*_2_ be the respective *ABFs* for traits 1 and 2, and let *M* be the maximum *ABF* observed at any SNP, for either trait. A Bayesian association metric for pruning may then be defined by

$$\begin{array}{ccc} ABF^{*} & = & max\left(ABF_{1},ABF_{2}\right)\\ & & +\;M\times I\{ABF_{1}>PO/R\;and\;ABF_{2}>PO/R\}, \end{array}$$

where *I*(*E*) is the indicator function, taking on value 1 when event *E* = {*ABF*_1_ > *PO*/*R* and *ABF*_2_ > *PO*/*R*} holds and 0, otherwise. When selecting between one of two SNPs in LD to remove, the form of ABF* increases the chance of retaining an SNP that has evidence of association with both traits, rather than an SNP that has high evidence strength for one trait, but little evidence for the other trait.

The analogous form for *P*‐values takes a slightly different form as follows:

$$P^{*}=\text{min}\left(P_{1},P_{2}\right)-1\times I\left\{P_{1}<\alpha \;and\;P_{2}<\alpha \right\},$$

where *P_1_* and *P*_2_ are the respective *P*‐values for traits 1 and 2, at a given SNP. Although P* is not a proper probability, it serves the purpose of maximizing the retention of SNPs that have sufficiently small *P*‐values for both traits.

SNPs are then ordered by decreasing ABF* (or increasing P*) for the selected trait and any SNP within 500 kb of the first SNP and in LD (*r* ^2^ > 0.1) with it is pruned out. Remaining SNPs are pruned out in a similar manner by continuing through the list of ordered SNPs. This is carried out using the clumping algorithm in PLINK version 1.07 [Purcell, 2009; Purcell et al., 2007].

The ABF* and P* are only used for pruning the data so that the SNPs are approximately independent, while simultaneously retaining SNPs that meet the significance threshold for both traits. Examination of association concordance between the traits is based on the individual *ABFs* (*ABF*_1_ and *ABF*_2_) and *P*‐values (*P*_1_ and *P*_2_) of the studies. In addition, as overlap SNPs are identified based on meeting the *ABF* (or *P*‐value) threshold for both traits, the direction of effect does not influence the overlap detection and may be the same or different among the traits.

### Test for Overlap Enrichment

We propose to test for more overlap than expected by chance between the genetic contributions to the two traits by examining the concordance between the levels of association evidence (high or low) at each SNP for the two traits. An SNP is considered to have high association evidence with trait *k* if *ABF_k_* > *PO*/*R* (referred to as high ABF) and low evidence otherwise (low *ABF*). This amounts to testing for SNP conditional independence between high (low) *ABF* of trait 1 and high (low) *ABF* of trait 2, where the association within each pair is conditional on the SNP. This is equivalent to testing for equal marginal frequencies between high (low) *ABF* of trait 1 and high (low) *ABF* of trait 2, as done by McNemar's mid‐*P* test [Fagerland et al., 2013]. McNemar's mid‐*P* test has been selected rather than McNemar's exact test because it has been shown that the mid‐*P* test has excellent power and only minor violations of significance level [Fagerland et al., 2013]. McNemar's test may be viewed as a paired version of a chi‐squared test.

The mid‐*P*‐value is calculated by constructing a matched‐pair contingency table (Table 1), based on the set of approximately independent SNPs.In this table, each SNP contributes to one of the cells according to the strength of association evidence for each trait, relative to the selected criteria (*R*, *π*_0_). For example, *n*_11_ is the number of SNPs that have *ABF* > *PO*/*R* for each of the traits 1 and 2, whereas *n*_10_ and *n*_01_ correspond to the counts of SNPs that are discordant with respect to the traits and high/low *ABF*. A similar table may be constructed for *P*‐values based on significance criteria *α*. The mid‐*P*‐value is given by

**Figure 1:**
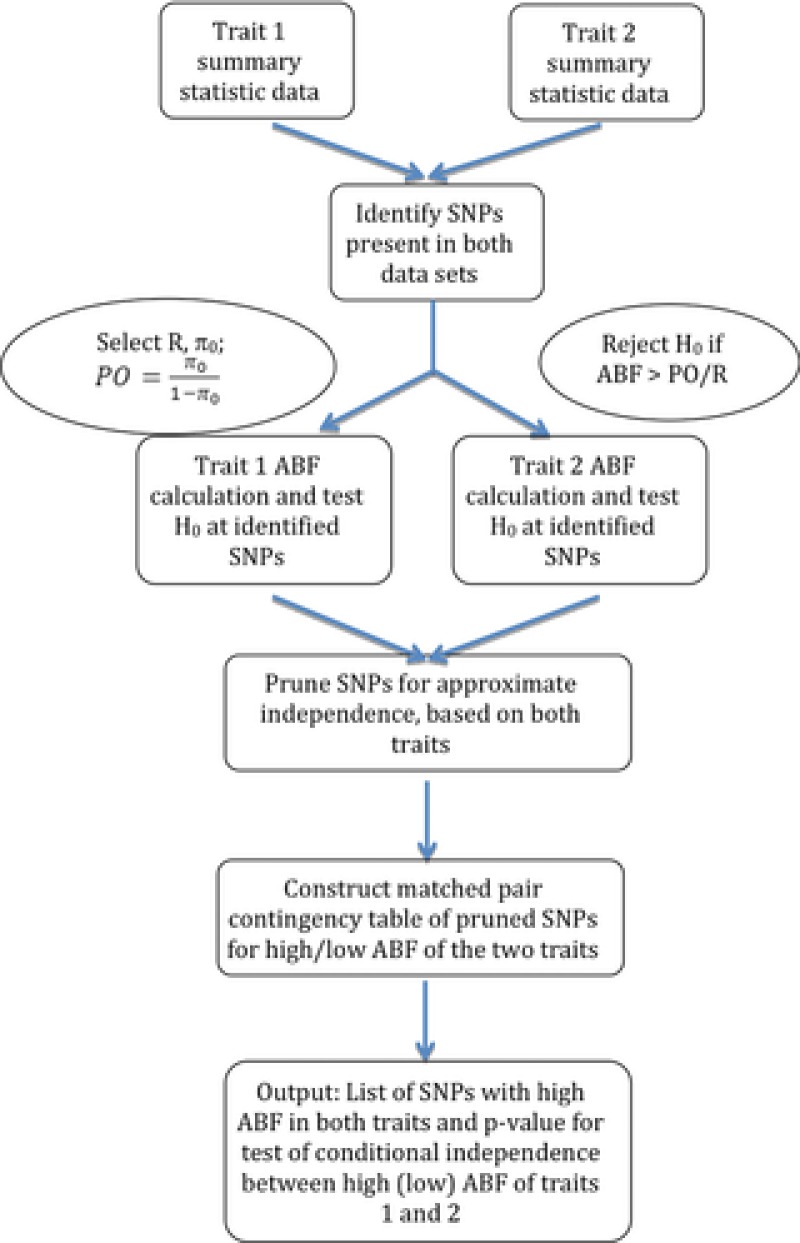
Overlap analysis flow chart.

2×∑x10=0minn10,n01fx10|n−f min (n10,n01)|n,

where the summation component is the McNemar exact conditional test one‐sided *P*‐value and *n* = *n*_01_ + *n*_10_, the total number of discordant SNPs. This differs from the χ^2^ contingency table analysis of Elliott et al. [2012], in which cells of the table corresponded to combinations of traits 1 and 2 (rows) with high and low *P*‐values (columns) and did not account for concordance/discordance at SNPs. A flow chart of the analysis steps proposed here is provided in Figure 1.

**Table 1:** **Matched‐pair contingency table for implementation of McNemar's test**

Trait 1\Trait 2	High *ABF* (*ABF* > *PO*/*R*)	Low *ABF* (*ABF* < *PO*/*R*)	
High *ABF* (*ABF* > *PO*/*R*)	*n_11_*	*n_10_*	
Low *ABF* (*ABF* < *PO*/*R*)	*n_01_*	*n_00_*	
			m

### Threshold Calibration

Overlap analyses may be completed using either Bayesian or frequentist approaches to measuring association significance. However, there does not exist a correspondence between *P*‐values and *ABFs* and a calibration between the two sets of thresholds is required in order to compare the performance of the approaches.

Because the Bayesian proportion of false positives (*PFP*) changes with sample size, there is no simple correspondence between thresholds from the two approaches. Thresholds for the Bayesian and frequentist approaches may be calibrated by matching the *PFP* resulting from each approach. PLINK version 1.07 [Purcell, 2009; Purcell et al., 2007] is used to simulate 5 million independent null SNPs from equal‐sized case‐control samples. As overlapping associated variants are to be identified within previous GWAS results, we focus on variants with *MAF* >0.05.

For a single GWAS with *n* cases, a calibrated *P*‐value threshold *α* is equal to the *PFP* for the selected Bayesian decision rule applied to null simulations with *n* cases. In practice a single threshold is applied to both studies of an overlap analysis, but a different calibrated *α *would be needed for each study to meet the Bayesian type I error rate. Therefore, we consider an upper *α*, *α*_U_,  defined as the *PFP* for the number of cases in the smaller study (less stringent for larger study) and a lower *α*, *α*_L_,   set as the *PFP* for the number of cases in the larger study (more stringent for smaller study). The lower *α* is applied to each study for overlap analysis, and likewise for the upper *α*. Conceptually, there is simplicity in applying the single *ABF* threshold to both studies, with an automatic adjustment of type I error rate according to study size. In contrast, the *P*‐value threshold dictates the type I error rate as identical, irrespective of sample size.

**Figure 2:**
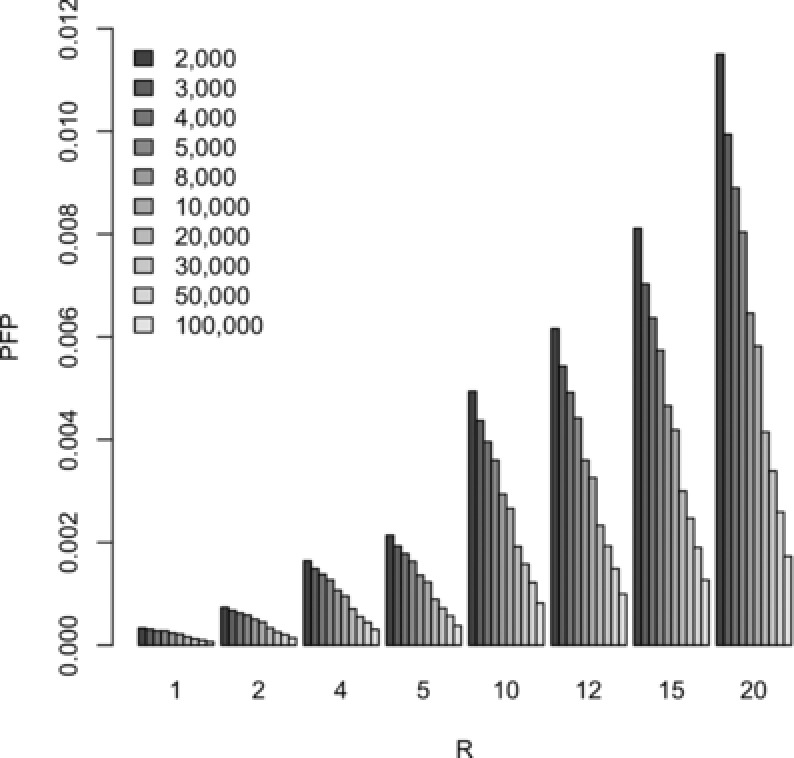
Type I error estimates for 5 million independent SNPs from equal‐sized case‐control samples (size in legend) using a Bayesian threshold with *π*_0_ = 0.99 and varying *R*.

The Bayesian threshold is calculated under assumptions of a prior association probability equal to 0.99 and 0.999, and at various levels of cost ratios *R*, ranging from 1 to 20. Ten different settings for equal‐sized case‐control samples of size 2,000 each up to 100,000 each are considered in the simulations (see Fig. 2 for increment details). The calibration tables are based on 1:1 case‐control ratios, which coincide with the simulation setup for the power studies. We also provide regression models, which may be used to extrapolate from this table to obtain thresholds for sample sizes that are not included in the table, as we illustrate for the power study involving studies of 15,000 each of cases and controls.

As the *PFP* for a given sample size and Bayesian threshold determines the analogous *P*‐value threshold for a study with a similar number of cases, we may extrapolate our *PFP* estimates to an alternative sample size by turning to regression. The type I error estimate may be approximated by a regression model of the –log_10_(*PFP*) against a quadratic function of log_10_(*N*), where *N* is the number of cases in the study. QQ plots of the standardized residuals suggest approximate normality, whereas plots comparing the fitted –log_10_(*PFP*) values and –log_10_(*PFP*) estimates against log_10_(*N*) suggest that the regression models appropriately fit the data. Examples of these plots are given in supplementary Figure S1 for *R* = 2, 15, and 20.

Although the calibration between approaches is based on a 1:1 case‐control ratio, we found that there was little difference in the results for case‐control ratios of 1:1.5 and 1:2, as in the arcOGEN and GIANT studies, respectively. In particular, the calibration based on *N* each of cases and controls was found to coincide well for case‐control ratios of 1:1.5 or 1:2 and *N* cases (numerical examples are provided in the Results). Therefore, the calibrations provided are likely to be good approximations for case‐control studies where there may be up to twice as many controls.

### Power Comparison

Power is compared between the frequentist and Bayesian approaches to detect a single SNP that is associated with two traits. The objective is to examine detection of overlap at a single SNP by each approach, and how the powers change with the *MAF* and effect sizes of the SNP in different studies for various sample size combinations.

As in the threshold calibration simulations, power approximations are based on 5 million independent SNPs. Various combinations of study sizes for overlap analysis are considered, where study *k* has *N_k_* each of cases and controls, and the sizes considered are 5,000; 10,000; 15,000; 20,000; and 30,000. For notational convenience we assume *N_1_ < N_2_*. At a shared causal variant, the *MAF* is either 0.1 or 0.2 and the *OR* for each trait is set to each possible combination of *OR* pairs involving 1.1 and/or 1.2. As the direction of effect does not affect the level of association evidence, we only consider the positive effect direction for both traits.

Bayesian thresholds are determined based on *π*_0_ = 0.99 or 0.999 and eight values of *R* ranging from 1 to 20; an SNP is identified as associated with both traits if *ABF* > *PO*/*R* for both traits and the proportion of such SNPs estimates the power of overlap detection based on *ABFs*. *P*‐value levels of significance are selected for a given Bayesian decision rule according to Table 2 and supplementary Table S1, based on *R*, *π*_0_, and *N*_1_ (for upper *α*) or *N*_2_ (for lower α); the power for upper *α* is approximated by the number of SNPs having *P*‐value <*α*_U_ for both traits, while power for *α*_L_ is defined in a similar manner.

### Description of Datasets

In the GIANT Extremes obesity meta‐analysis, obesity class I cases were defined as individuals who have *BMI* ≥30 kg/m^2^, while controls have *BMI* <25 kg/m^2^. The arcOGEN data had been imputed using the 1000 Genomes CEU haplotypes from the 2010 interim release in NCBI build 37 (hg19) coordinates [The 1000 Genomes Project Consortium, 2010], whereas GIANT made use of the haplotypes from the Phase II HapMap CEU population (build 36) [The International HapMap Consortium, 2003]. Due to both studies containing the 1958 Birth Cohort among the control samples, this cohort was excluded from the GIANT meta‐analysis. We then used the LiftOver tool (http://genome.sph.umich.edu/wiki/LiftOver) in order to bring the GIANT data to build 37.

The GIANT study excluding the 1958 Birth Cohort consists of 32,142 cases and 64,461 controls, whereas arcOGEN has 7,410 cases, and 11,009 controls. There were 2,087,589 SNPs present in both datasets that had *MAF* >0.05 in the 1000 Genomes CEU population. After LD pruning based on the association metric described in Materials and Methods, the number of SNPs included in the overlap analysis ranged from 88,980 to 91,122, depending on the threshold settings.

## Results

### Simulations: Threshold Calibration

Here, we empirically illustrate in single‐disease associations that *BFs* have the advantage over *P*‐values of a decreasing *PFP* as study size increases, whereas for *P*‐values the *PFP* fluctuates near the *P*‐value threshold *α *regardless of study size (as expected). The *PFP* at various *R* values under *π*_0_ = 0.99 is compared in Figure 2; Table 2 and supplementary Table S1 provide these type I error estimates under *π*_0_ = 0.99 and *π*_0_ = 0.999, respectively. There is a general trend of a 0.7‐fold increase in the exponent of the type I error estimates between samples having cases and controls each of size 2,000 and those having 100,000 for each.

**Table 2:** **Type I error estimates using a Bayesian threshold log_10_*θ*, determined from *π*_0_ = 0.99 and varying *R***

*N*\*R* (log_10_ *θ*)	1 (1.996)	2 (1.695)	4 (1.394)	5 (1.297)	10 (0.996)	12 (0.916)	15 (0.820)	20 (0.695)
2,000	3.33 × 10^–4^	7.38 × 10^–4^	1.64 × 10^–3^	2.14 × 10^–3^	4.94 × 10^–3^	6.16 × 10^–3^	8.11 × 10^–3^	1.15 × 10^–2^
3,000	3.10 × 10^–4^	6.74 × 10^–4^	1.49 × 10^–3^	1.93 × 10^–3^	4.37 × 10^–3^	5.43 × 10^–3^	7.03 × 10^–3^	9.94 × 10^–3^
4,000	2.77 × 10^–4^	6.24 × 10^–4^	1.38 × 10^–3^	1.78 × 10^–3^	3.96 × 10^–3^	4.92 × 10^–3^	6.37 × 10^–3^	8.90 × 10^–3^
5,000	2.77 × 10^–4^	5.84 × 10^–4^	1.27 × 10^–3^	1.63 × 10^–3^	3.60 × 10^–3^	4.42 × 10^–3^	5.74 × 10^–3^	8.04 × 10^–3^
8,000	2.34 × 10^–4^	5.05 × 10^–4^	1.07 × 10^–3^	1.36 × 10^–3^	2.94 × 10^–3^	3.60 × 10^–3^	4.66 × 10^–3^	6.46 × 10^–3^
10,000	2.16 × 10^–4^	4.51 × 10^–4^	9.56 × 10^–4^	1.23 × 10^–3^	2.66 × 10^–3^	3.26 × 10^–3^	4.19 × 10^–3^	5.82 × 10^–3^
15,000*	1.79 × 10^–4^	3.78 × 10^–4^	8.05 × 10^–4^	1.03 × 10^–3^	2.22 × 10^–3^	2.70 × 10^–3^	3.47 × 10^–3^	4.81 × 10^–3^
20,000	1.61 × 10^–4^	3.37 × 10^–4^	7.07 × 10^–4^	8.94 × 10^–4^	1.92 × 10^–3^	2.33 × 10^–3^	3.00 × 10^–3^	4.15 × 10^–3^
30,000	1.22 × 10^–4^	2.55 × 10^–4^	5.54 × 10^–4^	7.19 × 10^–4^	1.58 × 10^–3^	1.93 × 10^–3^	2.47 × 10^–3^	3.39 × 10^–3^
50,000	9.62 × 10^–5^	2.04 × 10^–4^	4.44 × 10^–4^	5.71 × 10^–4^	1.22 × 10^–3^	1.49 × 10^–3^	1.90 × 10^–3^	2.59 × 10^–3^
100,000	6.72 × 10^–5^	1.39 × 10^–4^	3.05 × 10^–4^	3.79 × 10^–4^	8.19 × 10^–4^	9.96 × 10^–4^	1.27 × 10^–3^	1.73 × 10^–3^

Estimates are based on 5 million independent SNPs from equal‐sized case‐control samples, each of size *N*. Estimates at *N* = 15,000 are the result of a regression at each *R* value of the *PFP* estimates against a quadratic of log_10_ *N*.

To put these PFPs into perspective, we focus on the simulation results for case‐control studies consisting of 8,000 each and 30,000 each, which are respectively comparable to the arcOGEN and GIANT (excluding 1958 Birth Cohort) studies, as described in Materials and Methods. For example, when *π*_0_ = 0.99, *R* = 4, the type I error estimates for 8,000 each of cases and controls and for an arcOGEN‐sized study are 1.07 × 10^–3^ (Table 2) and 1.01 × 10^–3^, respectively. Likewise, at the same Bayesian threshold settings the PFPs for case‐control samples of 30,000 each and for a GIANT (excluding 1958 Birth Cohort)‐sized study are 5.54 × 10^–4^ (Table 2) and 4.62 × 10^–4^, respectively. Upon examination of Table 2 and supplementary Table S1, it is apparent that for any *R* setting at either *π*_0_ = 0.99 or 0.999, the Bayesian type I error estimate based on 8,000 cases is twice that of the 30,000 cases. For instance, at *π*_0_ = 0.99, *R* = 2, the *PFPs* are 5.05 × 10^–4^ and 2.55 × 10^–4^ for case samples of size 8,000 and 30,000, respectively (Table 2).

When the number of cases is different than the settings considered in the simulations, we use a regression model to determine the analogous *P*‐value threshold for a given Bayesian threshold. The general regression for each parameter setting of *R* and *π*_0_ takes the form −log10(PFP)=β0+β1 lo g10N+β2( lo g10N)2, where *N* is the number of cases in the study, and occasionally the linear term is removed from the final fitted model, as it is not statistically significant at level 0.05. The coefficient estimates and their standard errors from each of the fitted models are provided in supplementary Table S2, for *π*_0_ = 0.99, 0.999 and a range of *R* values. An estimate of –log_10_(*PFP*) for specific values of *π*_0_ and *R* may then be found for a certain number of cases *N* by referring to the appropriate fitted model and using the coefficient estimates from supplementary Table S2. This is illustrated for case‐control samples of 15,000 each, and *PFP* estimates at *π*_0_ = 0.99 and *π*_0_ = 0.999, for a range of cost ratios *R*, which are provided in Table 2 and supplementary Table S1.

### Simulations: Power Comparison

**Figure 3:**
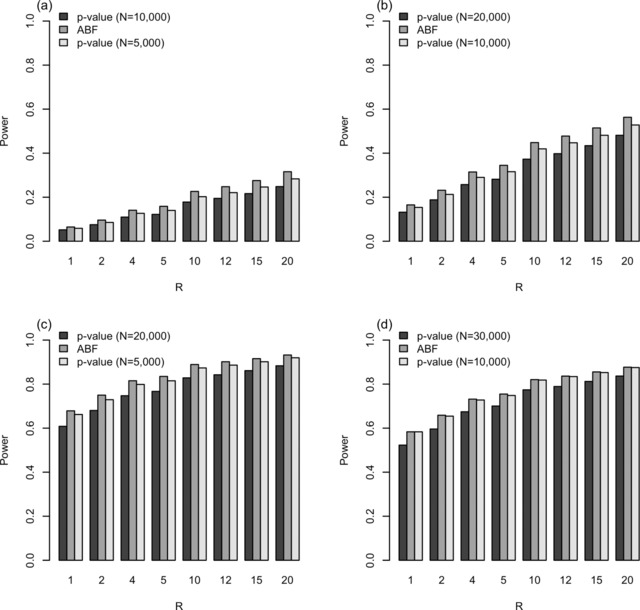
Power comparison for overlap analysis of two studies for various scenarios. (a) Study 1 has 5,000 each of cases and controls, whereas study 2 has 10,000 each. The causal SNP has *MAF* 0.1 and in studies 1 and 2, *OR* = 1.1 and *OR* = 1.2, respectively. (b) Study 1 has 10,000 each of cases and controls, whereas study 2 has 20,000 each. The causal SNP has *MAF* 0.1 and *OR* = 1.1 in both studies. (c) Study 1 has 5,000 each of cases and controls, whereas study 2 has 20,000 each. The causal SNP has *MAF* 0.1 and *OR* = 1.2 in both studies. (d) Study 1 has 10,000 each of cases and controls, whereas study 2 has 30,000 each. The causal SNP has *MAF* 0.2 and *OR* = 1.1 in both studies.

Power is compared to detect a single SNP that is associated with two traits, and it is clear that the maximum power is bounded by the minimum power between the two marginal studies. Representative examples from the power comparisons are displayed in Figure 3, for which detailed results may be found in supplementary Table S3. In addition, the results for a variety of simulation scenarios are given for thresholds based on *R* = 20 and *π*_0_ = 0.99 in supplementary Table S5. The Bayesian approach consistently attains a higher power than the frequentist method based on the lower *P*‐value threshold (from larger study), which is too stringent for the smaller‐sized study (see Fig. 3 and supplementary Tables S3–S5). Despite the upper *P*‐value threshold (from smaller study), upper *α*,  being slightly lenient for the larger study, the Bayesian approach tends to attain at least the same power (Fig. 3a–d, supplementary Table S3a–d).

In general, scenarios that tend to be underpowered (i.e., low *MAF* and small effect size) display a higher power gain (up to 4%) for the *ABF* implementation over the upper *P*‐value threshold (e.g., *MAF* 0.1; Fig. 3a and b, supplementary Tables S3a and b) and S4), whereas those that are high‐powered perform equally well (e.g., *MAF* 0.2; Fig. 3d, supplementary Tables S3d and S4). Also, the Bayesian power gain tends to increase with the ratio of the number of cases between the studies (or ratio of cases and controls, because we assume a 1:1 case‐control ratio). At lower *MAF* causal variants (e.g., *MAF* 0.1), the *P*‐value approach with threshold *α*_U_ either has a lower power than the *ABF* approach or is greater by a negligible amount (<0.5%; see Fig. 3a–c and supplementary Tables S3a–c and S4).

Among the scenarios considered, the one setting that displays a slight power gain (∼2%) for the frequentist over the Bayesian is in a high‐power setting (*MAF* 0.2) in which the effect size is larger in the smaller sample (*OR* 1.2 for smaller sample, *OR* 1.1 for larger sample); see supplementary Table S5. However, this gain in using the upper *α* approach is only observed when the smaller study is at most 5,000 each and the larger study has 10,000 each, and the gain dissipates with sample sizes beyond 15,000 (supplementary Table S5).

As a single overlap SNP is assumed in each of the 5 million replications, among these true association signals detected by *ABFs* or *P*‐values (the set of SNPs denoted $ABF\cup \alpha_{U}$) we compare the proportion of signals detected by *ABFs* that are not identified by *P*‐values and vice versa. These conditional proportions indicate that despite similar power differences between ABF and *P*‐value approaches, the higher‐powered method does not catch a similarly larger proportion of variants than the other; when the ABF approach is higher powered, conditional proportions for *ABF*‐only detections are larger than conditional proportions for *P*‐value‐only detections when *P*‐values have higher power than *ABFs*.

For two studies consisting of equal‐sized case‐control samples of sizes 10,000 each and 20,000 each, with a shared causal variant having *MAF* 0.1 and *OR* 1.1, *ABFs* identify approximately 99% of the variants detected by either method, based on *π*_0_ = 0.99 or 0.999, whereas *P*‐values identify 92–93% of the variants when *π*_0_ = 0.99 and as little as 89.4% when *R* = 2, *π*_0_ = 0.999 (*π*_0_ = 0.99 results in supplementary Table S6; *π*_0_ = 0.999 not shown). For example, at *R* = 2, *π*_0_ = 0.99 the power advantage with *ABFs* is a 1.9% increase (supplementary Table S3), but 8.4% (97,304/1,160,779) of the detected signals are found only by *ABFs*, whereas the reverse proportion is 0.26% for signals detected only by *P*‐values (supplementary Table S6).

In contrast, when the causal variant has *MAF* 0.2 in studies consisting of 5,000 each of cases and controls (*OR* 1.2) and 10,000 each (*OR* 1.1), the *P*‐value approach has a general power gain of 2% over *ABFs* (supplementary Table S5), and the conditional proportions indicate that *P*‐values only detect 2–4% more variants than *ABFs* (supplementary Table S6). For instance, at *R* = 2, *π*_0_ = 0.99, the *P*‐value approach is higher powered by 1.9% (supplementary Table S5), yet 3% (100,566/3,308,889) of the identified signals are found only by *P*‐values, and the complementary proportion for ABF‐only‐detected signals is 0.17% (supplementary Table S6). Similar behavior is observed for the overlap analysis of studies consisting of 5,000 each and 15,000 each, with the proportion of variants detected only by *P*‐values ranging from 1% to 3% (supplementary Table S6).

### Application: Obesity and Osteoarthritis

The proposed methods were applied to the overlap analysis of obesity (GIANT Extremes meta‐analysis [Berndt et al., 2013]) and knee and/or hip osteoarthritis (arcOGEN GWAS [arcOGEN Consortium et al., 2012]) to identify SNPs associated with both traits, as well as test for an excess of more shared signals than expected by chance. This was completed using summary statistics from the original GIANT meta‐analysis, as well as those based on the exclusion of the 1958 Birth Cohort. As the two sets of results are quite similar, we report only those based on the latter, which did not encounter the issue of overlapping control sets between the arcOGEN and GIANT datasets.

Concordance between the full GIANT study and that with the exclusion of the 1958 Birth Cohort is near 0.99, indicating that the reduction in sample size has little impact on this large meta‐analysis. Specifically, in comparing all SNPs with *MAF* >0.05, the Pearson correlation coefficients for log_10_ *ABF* and –log_10_(*P*‐value) are 0.991 and 0.988, respectively, whereas the respective measures are 0.996 and 0.995 when the concordance is measured for the set of common SNPs with a *P*‐value <0.01 in the full GIANT meta‐analysis.

**Figure 4:**
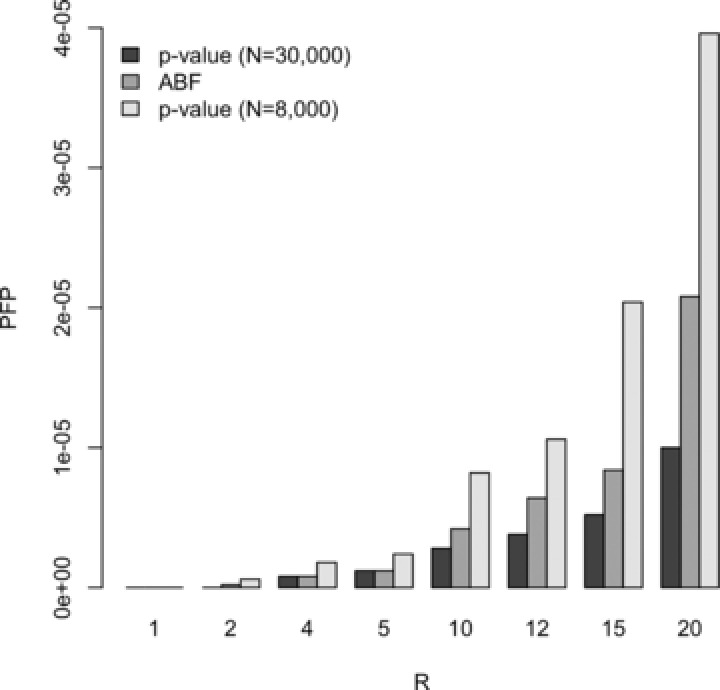
Overlap type I error estimates for 5 million independent SNPs from studies of the same size as arcOGEN and GIANT (excluding 1958 Birth Cohort).

Based on the sample sizes of the GIANT study excluding the 1958 Birth Cohort and of the arcOGEN study, type I error estimates for the overlap analysis were obtained via a simulation study of 5 million independent SNPs and are compared in Figure 4 for the set of Bayesian decision rules with *π*_0_ = 0.99. As in the examination of type I error to detect an association at a single variant in a single study, the marginal type I error estimate for the Bayesian approach is smaller for the larger of the two studies.

The sets of SNPs identified by each method are not always overlapping and additional signals are often in already detected genes. As pruning was performed separately for *ABFs* and *P*‐values, within the merged list of 80 overlap SNPs identified by *ABFs* (*π*_0_ = 0.99, *R* = 20) and/or *P*‐values (0.0065), there were 15 pairs of SNPs in the same LD clump (*r* ^2^ >0.1 and within 500 kb). The level of LD was then determined for each pair of such SNPs via the software SNAP (SNP Annotation and Proxy Search [Johnson et al., 2008]). As the lowest LD measurement was 0.57 for these pairs, the SNP with the smaller ABF was removed from the pair, resulting in a list of 65 approximately independent SNPs.

The top 20 independent signals that have been identified by each method are provided in Table 3, together with the assigned rank from each method, and the nearest gene. Genes that have previously been identified as containing SNPs that are genome‐wide significantly associated with obesity‐related phenotypes (Ensembl; http://www.ensembl.org/index.html) are labeled with a double asterisk in Table 3, whereas those that have been observed as highly significant (*P*‐value < 9 × 10^–5^) have a single asterisk.

**Table 3:** **Top 20 independent signals by Bayesian and frequentist assessment, ranked by each of the *ABF* and *P*‐value approaches**

*ABF* rank	*P*‐value rank	Chromosome	SNP rsid	Min. ABF	Max. *P*‐value	Nearest gene
1	1	16	rs7185735	2.741	3.20 × 10^–5^	FTO**
2	2	2	rs7607584	1.899	3.82 × 10^–4^	LRP1B**
3	4	8	rs11986122	1.166	1.63 × 10^–3^	*MSRA*
4	5	1	rs1834527	1.111	1.83 × 10^–3^	NEGR1**
5	7	15	rs11853080	1.088	2.01 × 10^–3^	ACSBG1*
6	15	16	rs17218700	1.034	3.28 × 10^–3^	FTO**
7	3	3	rs1355782	1.004	1.44 × 10^–3^	CPNE4**
8	9	6	rs4714924	0.992	2.47 × 10^–3^	*RCAN2*
9	12	16	rs3848299	0.990	2.91 × 10^–3^	FTO**
10	10	8	rs6601560	0.981	2.49 × 10^–3^	*XKR6*
11	29	4	rs13107325	0.969	4.41 × 10^–3^	SLC39A8/ZIP8**
12	18	1	rs6425451	0.925	3.41 × 10^–3^	SEC16B**
13	6	3	rs17530358	0.911	1.90 × 10^–3^	SRGAP3**
14	16	20	rs6063765	0.909	3.29 × 10^–3^	*ZFP64*
15	14	6	rs655370	0.900	3.05 × 10^–3^	RGS17*
16	61	3	rs3732869	0.889	7.28 × 10^–3^	*RASA2*
17	20	15	rs12441823	0.888	3.65 × 10^–3^	MAP2K5**
18	19	11	rs4514364	0.871	3.55 × 10^–3^	*LGR4*
19	31	3	rs6788477	0.846	4.75 × 10^–3^	–
20	21	22	rs5995843	0.842	3.71 × 10^–3^	*TNRC6B*
21	8	15	rs8024948	0.835	2.34 × 10^–3^	*CHSY1*
28	11	6	rs2395754	0.772	2.50 × 10^–3^	*OARD1, APOBEC2*
43	13	20	rs6072602	0.703	2.98 × 10^–3^	PTPRT*
47	17	4	rs17789621	0.671	3.35 × 10^–3^	*HERC6*

Also provided, are the minimum *ABF* and maximum *P*‐value between the two traits at each SNP. Genes with prior genome‐wide significant associations (*P* < 5 × 10^–8^) with obesity, *BMI*, and/or weight are denoted by a double asterisk (**), whereas those with previous highly significant associations (*P* < 9 × 10^–5^) are denoted by a single asterisk (*).

For both *ABFs* and *P*‐values, the strongest evidence of an overlap association with both obesity and osteoarthritis is a variant in *FTO*, which is unequivocally associated with adiposity [Fawcett and Barroso, 2010]. This variant is also associated with OA, as it is in high LD with both index SNPs in *FTO* that had been identified by Elliott et al. [2012] (*r* ^2^ = 0.838 with rs12149832) and Panoutsopoulou et al. [2013] (*r* ^2^ = 0.605 with rs8044769), suggesting that they are part of the same signal. Furthermore, two additional independent *FTO* variants are identified as associated with both obesity and OA, and both variants are ranked higher by *ABFs* rather than *P*‐values (see Table 3).

For the Bayesian and frequentist approaches, there is 80% agreement in the variants identified within the top five signals, as well as within the top 10 and 20 signals. Among the top 20 *ABF* signals, half are within/near genes known to have prior genome‐wide significant associations with obesity, *BMI*, and/or weight, while the *P*‐value approach assigns rank 29 to one of these signals (rs13107325 in *SLC39A8*/*ZIP8*, a zinc transporter).

We also tested if the number of detected overlap SNPs at various thresholds is more than expected by chance, and display these counts together with their McNemar mid‐*P*‐values in Table 4 (π_0_ = 0.99) and supplementary Table S3 (*π*_0_ = 0.999). When *π*_0_ = 0.99, there is a clear trend of more significant *P*‐values for the *ABF* analysis route, whereas the frequentist route counts are not considered to be different from chance at significance level 0.05 at *R* = 1 for both *P*‐value thresholds, as well as at *R* = 2 and 4 for the lower *α* threshold.

For a given set of threshold settings (*α*_L_, log_10_ *θ*, *α*_U_), where *θ* = *PO*/*R*, there is a tendency for an ordering of the counts of identified overlap variants with the number based on ABF strength being between those based on each of the two *P*‐value thresholds (Table 4). For example, when *π*_0_ = 0.99 and *R* = 10, so that log_10_ *θ* = 0.996, there are nine overlapping variants identified. This falls between the counts of overlapping variants identified by the corresponding *P*‐value thresholds: *α*_L_ = 0.0016 and *α*_U_  = 0.003, respectively, detect 6 and 15 shared associations.

**Table 4:** **Number of overlap variants identified by the *ABF* (log_10_*θ* threshold; *θ* = *PO*/*R*) at various *R* values with *π*_0_ = 0.99 and the corresponding lower and upper *P*‐value thresholds, based on 30,000 and 8,000 cases, respectively**

*ABF*			Lower *α*		Upper *α*	
*R*	log_10_ *θ* threshold	Number detected (McNemar mid‐*P*‐value)	*α*_L_ threshold (*N* = 30,000)	Number detected (McNemar mid‐*P*‐value)	α_U_ threshold (*N* = 8000)	Number detected (McNemar mid‐*P*‐value)
1	1.996	2 (1.06 × 10^–3^)	1.20 × 10^–4^	2 (1.53 × 10^–1^)	2.40 × 10^–4^	2 (6.95 × 10^–1^)
2	1.695	2 (4.28 × 10^–10^)	2.50 × 10^–4^	2 (6.98 × 10^–1^)	5.10 × 10^–4^	2 (3.55 × 10^–2^)
4	1.394	3 (4.20 × 10^–25^)	5.50 × 10^–4^	2 (6.38 × 10^–2^)	1.10 × 10^–3^	3 (2.11 × 10^–6^)
5	1.297	3 (7.89 × 10^–34^)	7.20 × 10^–4^	2 (3.37 × 10^–2^)	1.40 × 10^–3^	4 (3.22 × 10^–9^)
10	0.996	9 (1.16 × 10^–82^)	1.60 × 10^–3^	6 (1.92 × 10^–11^)	3.00 × 10^–3^	15 (2.22 × 10^–21^)
12	0.916	15 (2.77 × 10^–97^)	1.90 × 10^–3^	7 (1.90 × 10^–12^)	3.60 × 10^–3^	23 (5.75 × 10^–29^)
15	0.820	25 (5.77 × 10^–121^)	2.50 × 10^–3^	12 (1.86 × 10^–18^)	4.70 × 10^–3^	31 (3.63 × 10^–35^)
20	0.695	45 (1.95 × 10^–157^)	3.40 × 10^–3^	19 (9.24 × 10^–26^)	6.50 × 10^–3^	59 (1.95 × 10^–44^)

The McNemar *P*‐value follows the counts of detected overlap variants.

## Discussion

The use of BFs, rather than *P*‐values, allows an automatic adjustment of smaller type I error rate for larger samples (higher powered tests) for a fixed ABF threshold; for a fixed *P*‐value threshold, tests based on *P*‐values have identical type I error rates regardless of sample size (and power of the test). In the overlap analysis of two studies with different powers, this *ABF* approach simplifies the selection of a threshold for use in both studies, rather than choosing a *P*‐value threshold that is either too lenient for the larger study or too strict for the smaller study.

For the detection of variants associated with two traits, we made extensive comparisons between association strength assessed by BFs and by *P*‐values. These evaluations focus on identifying shared associations at the SNP level irrespective of any direction of effect. In an overlap analysis of studies consisting of different sample sizes, the Bayesian approach had a consistent power advantage over the more stringent *P*‐value threshold (calibrated for larger sample), and a tendency to attain at least the power of the more lenient *P*‐value threshold (calibrated for smaller sample).

We provide a calibration table between *ABFs* and *P*‐values for a range of sample sizes, as well as a simple means of estimating a *P*‐value threshold coinciding with a particular Bayesian threshold rule *(π_0_, R)* for a certain sample size. As BFs have less intuition behind them than *P*‐values, for a selected Bayesian threshold rule, the tables or regression models may serve as a reference to the coinciding *P*‐value threshold. Therefore, in applying a single Bayesian threshold for each sample set of an overlap analysis the tables may be used to determine the approximate false‐positive rate within each sample set, and thus removing some of the opaqueness of Bayesian approaches. Alternatively, if a certain *PFP* is desired, the table and models may aid in selection of the Bayesian threshold parameters.

In our overlap analysis of obesity and osteoarthritis, a variant in *FTO*, which is established as associated with both traits, was the top signal based on both *ABFs* and on *P*‐values, which demonstrates the validity of our approach. There were several additional signals within the top 20 for either approach that are within established obesity loci, though not for OA. However, rs6788477, which was rank 19 for ABFs and rank 31 for *P*‐values, is 6.79 MB from *GNL3*, an established OA locus. In addition, we detected an obesity‐associated SNP, rs13107325 (ABF rank 11, *P*‐value rank 29) in the gene *SLC39A8/ZIP8*, which has been strongly implicated in OA pathogenesis [Kim et al., 2014]. As it is unknown for all identified SNPs outside of *FTO* whether or not there is a true association with both obesity and OA, there was difficulty in comparing the *ABF* and *P*‐value approaches. This was overcome by considering conditional probabilities in our simulation studies.

In simulation studies under the alternative hypothesis, we considered the probability that the *ABF* approach identified a signal, given that this signal was identified by at least one of the methods. Likewise, the analogous probability was examined for *P*‐values. We found that in scenarios of similar power differences between the approaches, the *ABF* approach was able to capture a higher proportion of overlapping associated variants than *P*‐values.

Although Bayesian approaches are sometimes considered less appealing than frequentist, there is a clear advantage when a single threshold is to be used for multiple studies. In particular, the type I error rate is appropriately adjusted for a given Bayesian threshold, such that the type I error is smaller for the larger, more powerful study. The *ABF* route lends simplicity in threshold selection for studies of different sizes, as the *ABF* is directly comparable between two studies irrespective of the study size. In contrast, a relatively small *P*‐value does not have the same meaning in studies of very different sizes.

## Supporting information


**Figure S1**: Standardised residual QQ‐plots to assess normality are provided in the left panel for the regression models with R=2 (a), R=15 (c) and R=20 (e). The right panel displays corresponding plots of the estimated values (from simulations) of –log_10_(PFP) against log_10_(N) with the fitted –log_10_(PFP) regression line for R=2 (b), R=15 (d) and R=20 (f).
**Table S1**: Type I error estimates using a Bayesian threshold log_10_θ, determined from π_0_ = 0.999 and varying R. Estimates are based on 5 million independent SNPs from equal‐sized case‐control samples, each of size N. Estimates at N=15,000 are the result of a regression at each R value of the PFP estimates against a quadratic of log_10_N.
**Table S2**: Regression coefficient estimates from model $\log_{10}\left(PFP\right)=\beta_{0}+\beta_{1}\log_{10}N+\beta_{2}{\left(\log_{10}N\right)}^{2}$ together with their standard errors σ_i_. All coefficient estimates have p‐value < 0.01.
**Table S3**: Overlap power results coinciding with Figure 3, where N_i_ denotes the sample size for each of the cases and controls in study *i*, OR_i_ denotes the OR of the causal variant in study *i*, which has the specified MAF
**Table S4**: Overlap power results based on R=20 and π_0_=0.99 for the ABF threshold and coinciding p‐value threshold (from Table 2) , where N_i_ denotes the sample size for each of the cases and controls in study *i*, OR_i_ denotes the OR of the causal variant in study *i*, which has MAF 0.1.
**Table S5**: Overlap power results for the scenario where the causal variant has MAF 0.2 and a larger effect in the smaller sample: OR_1_ = 1.2 and OR_2_ = 1.1. The sample size of each of the cases and controls in study *i*, is denoted by N_i._
**Table S6**: Probabilities conditional on overlap SNPs detected by ABF or upper α, α_U_ for various values of R and π_0_=0.99 where probabilities conditional on this set of SNPs are denoted *P^C^*.
**Table S7**: Number of overlap variants identified by the ABF (log_10_θ threshold), according to various R values with π_0_=0.999 and the corresponding lower and upper p‐value thresholds, based on 30,000 and 8,000 cases, respectively. The McNemar p‐value follows the counts of detected overlap variants.
