# Supplementary material for: A Bayesian Approach to the Overlap Analysis of Epidemiologically Linked Traits
Source: Genet Epidemiol. 2015 Sep 28;39(8):624–34. doi: 10.1002/gepi.21919 (PMC4832282; doi:10.1002/gepi.21919)
Supplement: Supplementary file 1 — Figure S1: Standardised residual QQ‐plots to assess normality are provided in the left panel for the regression models with R=2 (a), R=15 (c) and R=20 (e). The right panel displays corresponding plots of the estimated values (from simulations) of –log10(PFP) against log10(N) with the fitted –log10(PFP) regression line for R=2 (b), R=15 (d) and R=20 (f). Table S1: Type I error estimates using a Bayesian threshold log10θ, determined from π0 = 0.999 and varying R. Estimates are based on 5 million independent SNPs from equal‐sized case‐control samples, each of size N. Estimates at N=15,000 are the result of a regression at each R value of the PFP estimates against a quadratic of log10N. Table S2: Regression coefficient estimates from model log10(PFP)=β0+β1log10N+β2(log10N)2 together with their standard errors σi. All coefficient estimates have p‐value < 0.01. Table S3: Overlap power results coinciding with Figure 3, where Ni denotes the sample size for each of the cases and controls in study i, ORi denotes the OR of the causal variant in study i, which has the specified MAF Table S4: Overlap power results based on R=20 and π0=0.99 for the ABF threshold and coinciding p‐value threshold (from Table 2) , where Ni denotes the sample size for each of the cases and controls in study i, ORi denotes the OR of the causal variant in study i, which has MAF 0.1. Table S5: Overlap power results for the scenario where the causal variant has MAF 0.2 and a larger effect in the smaller sample: OR1 = 1.2 and OR2 = 1.1. The sample size of each of the cases and controls in study i, is denoted by Ni. Table S6: Probabilities conditional on overlap SNPs detected by ABF or upper α, αU for various values of R and π0=0.99 where probabilities conditional on this set of SNPs are denoted PC. Table S7: Number of overlap variants identified by the ABF (log10θ threshold), according to various R values with π0=0.999 and the corresponding lower and upper p‐value thresholds, based on 30,000 and 8,000 cas [file GEPI-39-624-s001.docx]

**Supplementary Material**

**Consortia Details**

**The arcOGEN Consortium: Arthritis Research UK Osteoarthritis Genetics Consortium**

John Loughlin^1^, Nigel Arden^2,3^, Fraser Birrell^1^, Andrew Carr^2,4^, Kay Chapman^5^, Panos Deloukas^6^, Michael Doherty^7^, Andrew McCaskie^1^, William E. R. Ollier^8^, Ashok Rai^9^, Stuart H. Ralston^10^, Timothy D. Spector^11^, Ana M. Valdes^12^, Gillian A. Wallis^13^, J. Mark Wilkinson^14^, Eleftheria Zeggini^15^

1. Musculoskeletal Research Group, Institute of Cellular Medicine, Newcastle University, Newcastle upon Tyne, UK
2. Oxford NIHR Musculoskeletal Biomedical Research Unit, Nuffield Department of Orthopaedics, Rheumatology and Musculoskeletal Sciences, University of Oxford, Oxford, UK
3. MRC Lifecourse Epidemiology Unit and NIHR Nutrition Biomedical Research Centre, University of Southampton, Southampton, UK
4. Botnar Institute of Musculoskeletal Sciences, Nuffield Department of Orthopaedics, Rheumatology and Musculoskeletal Sciences, University of Oxford, Oxford, United Kingdom
5. NIHR Biomedical Research Unit and ARUK Centre of excellence for Sport, Exercise and Osteoarthritis, University of Oxford, Oxford, UK
6. William Harvey Research Institute, Barts and The London School of Medicine and Dentistry, Queen Mary University of London, London, UK
7. Academic Rheumatology, University of Nottingham, Nottingham, UK
8. Centre for Integrated Genomic Medical Research, University of Manchester, Manchester, UK
9. Worcestershire Acute Hospitals NHS Trust, Worcester, UK.
10. Centre for Genomic and Experimental Medicine, Institute of Genetics and Molecular Medicine, University of Edinburgh, Edinburgh, UK.
11. Department of Twin Research and Genetic Epidemiology, King's College London, London, UK
12. Division of Rheumatology, Orthopaedics, and Dermatology, School of Medicine, University of Nottingham, Nottingham, UK
13. Wellcome Trust Centre for Cell-Matrix Research, University of Manchester, UK
14. Department of Human Metabolism, University of Sheffield, Sheffield, UK
15. Wellcome Trust Sanger Institute, Hinxton, Cambridge, UK

**The GIANT Consortium: Genetic Investigation of ANthropometric Traits Consortium**

Sonja I. Berndt**^1^**, Stefan Gustafsson**^2^**, Reedik Mägi**^3,4^**, Andrea Ganna**^2^***, Eleanor Wheeler**^5^**, Mary F. Feitosa**^6^**, Anne E. Justice**^7^**, Keri L. Monda**^7,8^**, Damien C. Croteau-Chonka**^9^**, Felix R. Day**^10^**, Tõnu Esko**^4,11^**, Tove Fall**^2^**, Teresa Ferreira**^3^**, Davide Gentilini**^12^**, Anne U. Jackson**^13^**, Jian'an Luan**^10^**, Joshua C. Randall**^3,5^**, Sailaja Vedantam**^14,15,16^**, Cristen J. Willer**^17,18,19^**, Thomas W. Winkler**^20^**, Andrew R. Wood**^21^**, Tsegaselassie Workalemahu**^22,23^**, Yi-Juan Hu**^24^**, Sang Hong Lee**^25^**, Liming Liang**^26,27^**, Dan-Yu Lin**^28^**, Josine L. Min**^3^**, Benjamin M. Neale**^29^**, Gudmar Thorleifsson**^30^**, Jian Yang**^31,32^**, Eva Albrecht**^33^**, Najaf Amin**^34^**, Jennifer L. Bragg-Gresham**^13^**, Gemma Cadby**^35,36,37^**, Martin den Heijer**^38^**, Niina Eklund**^39,39^**, Krista Fischer**^4^**, Anuj Goel**^40^**, Jouke-Jan Hottenga**^41^**, Jennifer E. Huffman**^42^**, Ivonne Jarick**^43^**, Åsa Johansson**^44,45^**, Toby Johnson**^46^**, Stavroula Kanoni**^5^**, Marcus E. Kleber**^47,48^**, Inke R. König**^49^**, Kati Kristiansson**^39^**, Zoltán Kutalik**^50,51^**, Claudia Lamina**^52^**, Cecile Lecoeur**^53,54^**, Guo Li**^55^**, Massimo Mangino**^56^**, Wendy L. McArdle**^57^**, Carolina Medina-Gomez**^34,58,59^**, Martina Müller-Nurasyid**^33,60,61^**, Julius S. Ngwa**^62^**, Ilja M. Nolte**^63^**, Lavinia Paternoster**^64^**, Sonali Pechlivanis**^65^**, Markus Perola**^4,39,66^**, Marjolein J. Peters**^34,58,59^**, Michael Preuss**^49,67^**, Lynda M. Rose**^68^**, Jianxin Shi**^1^**, Dmitry Shungin**^69,70,71^**, Albert Vernon Smith**^72,73^**, Rona J. Strawbridge**^74^**, Ida Surakka**^39,66^**, Alexander Teumer**^75^**, Mieke D. Trip**^76,77^**, Jonathan Tyrer**^78^**, Jana V. Van Vliet-Ostaptchouk**^79,80^**, Liesbeth Vandenput**^81^**, Lindsay L. Waite**^82^**, Jing Hua Zhao**^10^**, Devin Absher**^82^**, Folkert W. Asselbergs**^83^**, Mustafa Atalay**^84^**, Antony P. Attwood**^85^**, Anthony J. Balmforth**^86^**, Hanneke Basart**^76^**, John Beilby**^87,88^**, Lori L. Bonnycastle**^89^**, Paolo Brambilla**^90^**, Marcel Bruinenberg**^80^**, Harry Campbell**^91^**, Daniel I. Chasman**^68,92^**, Peter S. Chines**^89^**, Francis S. Collins**^89^**, John M. Connell**^93,94^**, William Cookson**^95^**, Ulf de Faire**^96^**, Femmie de Vegt**^97^**, Mariano Dei**^98^**, Maria Dimitriou**^99^**, Sarah Edkins**^5^**, Karol Estrada**^34,58,59^**, David M. Evans**^64^**, Martin Farrall**^40^**, Marco M. Ferrario**^100^**, Jean Ferrières**^101^**, Lude Franke**^80,102^**, Francesca Frau**^103^**, Pablo V. Gejman**^104,105^**, Harald Grallert**^106^**, Henrik Grönberg**^2^**, Vilmundur Gudnason**^72,73^**, Alistair S. Hall**^107^**, Per Hall**^2^**, Anna-Liisa Hartikainen**^108^**, Caroline Hayward**^42^**, Nancy L. Heard-Costa**^109^**, Andrew C. Heath**^110^**, Johannes Hebebrand**^111^**, Georg Homuth**^75^**, Frank B. Hu**^22^**, Sarah E. Hunt**^5^**, Elina Hyppönen**^112^**, Carlos Iribarren**^113^**, Kevin B. Jacobs**^1,114^**, John-Olov Jansson**^115^**, Antti Jula**^116^**, Mika Kähönen**^117^**, Sekar Kathiresan**^118,119,120^**, Frank Kee**^121^**, Kay-Tee Khaw**^122^**, Mika Kivimaki**^123^**, Wolfgang Koenig**^124^**, Aldi T. Kraja**^6^**, Meena Kumari**^123^**, Kari Kuulasmaa**^125^**, Johanna Kuusisto**^126^**, Jaana H. Laitinen**^127^**, Timo A. Lakka**^84,128^**, Claudia Langenberg**^10,123^**, Lenore J. Launer**^129^**, Lars Lind**^130^**, Jaana Lindström**^131^**, Jianjun Liu**^132^**, Antonio Liuzzi**^133^**, Marja-Liisa Lokki**^134^**, Mattias Lorentzon**^81^**, Pamela A. Madden**^110^**,Patrik K. Magnusson**^2^**, Paolo Manunta**^135^**, Diana Marek**^50,51^**, Winfried März**^48,136^**, Irene Mateo Leach**^137^**, Barbara McKnight**^138^**, Sarah E. Medland**^32^**, Evelin Mihailov**^4,11^**, Lili Milani**^4^**, Grant W. Montgomery**^32^**, Vincent Mooser**^139^**, Thomas W. Mühleisen**^140,141^**, Patricia B. Munroe**^46^**, Arthur W. Musk**^142,143,144^**, Narisu Narisu**^89^**, Gerjan Navis**^145^**, George Nicholson**^146,147^**, Ellen A. Nohr**^148^**, Ken K. Ong**^10,149^**, Ben A. Oostra**^59,150,151^**, Colin N.A. Palmer**^152^**, Aarno Palotie**^5,66^**, Ju-hyun Park^1^, John F. Peden**^153^**, Nancy Pedersen**^2^**, Annette Peters**^106,154,155^**, Ozren Polasek**^156^**, Anneli Pouta**^108,157^**, Peter P. Pramstaller**^158,159,160^**, Inga Prokopenko**^3,161^**, Carolin Pütter**^65^**, Aparna Radhakrishnan**^5,162,163^**, Olli Raitakari**^164,165^**, Augusto Rendon**^85,162,163,166^**, Fernando Rivadeneira**^34,58,59^**, Igor Rudan**^91^**, Timo E. Saaristo**^167,168^**, Jennifer G. Sambrook**^162,163^**, Alan R. Sanders**^104,105^**, Serena Sanna**^98^**, Jouko Saramies**^169^**, Sabine Schipf**^170^**, Stefan Schreiber**^171^**, Heribert Schunkert**^67,172^**, So-Youn Shin**^5^**, Stefano Signorini**^173^**, Juha Sinisalo**^174^**, Boris Skrobek**^53,54^**, Nicole Soranzo**^5,56^**, Alena Stančáková**^175^**, Klaus Stark**^176^**, Jonathan C. Stephens**^162,163^**, Kathleen Stirrups**^5^**, Ronald P. Stolk**^63,80^**, Michael Stumvoll**^177,178^**, Amy J. Swift**^89^**, Eirini V. Theodoraki**^99^**, Barbara Thorand**^154^**, David-Alexandre Tregouet**^179^**, Elena Tremoli**^180^**, Melanie M. Van der Klauw**^79,80^**, Joyce B.J. van Meurs**^34,58,59^**, Sita H. Vermeulen**^97,181^**, Jorma Viikari**^182^**, Jarmo Virtamo**^125^**, Veronique Vitart**^42^**, Gérard Waeber**^183^**, Zhaoming Wang**^1,114^**, Elisabeth Widén**^66^**, Sarah H. Wild**^91^**, Gonneke Willemsen**^41^**, Bernhard R. Winkelmann**^184^**, Jacqueline C.M. Witteman**^34,59^**, Bruce H.R. Wolffenbuttel**^79,80^**, Andrew Wong**^149^**, Alan F. Wright**^42^**, M. Carola Zillikens**^58,59^**, Philippe Amouyel**^185^**, Bernhard O. Boehm**^186^**, Eric Boerwinkle**^187^**, Dorret I. Boomsma**^41^**, Mark J. Caulfield**^46^**, Stephen J. Chanock**^1^**, L. Adrienne Cupples**^62^**, Daniele Cusi**^103,188^**, George V. Dedoussis**^99^**, Jeanette Erdmann**^67,172^**, Johan G. Eriksson**^189,190,191^**, Paul W. Franks**^69,70,192^**, Philippe Froguel**^53,54,193^**, Christian Gieger**^33^**, Ulf Gyllensten**^44^**, Anders Hamsten**^74^**, Tamara B. Harris**^129^**, Christian Hengstenberg**^176^**, Andrew A. Hicks**^158^**, Aroon Hingorani**^123^**, Anke Hinney**^111^**, Albert Hofman**^34,59^**, Kees G. Hovingh**^76^**, Kristian Hveem**^194^**, Thomas Illig**^106,195^**, Marjo-Riitta Jarvelin**^157,196,197,198^**, Karl-Heinz Jöckel**^65^**, Sirkka M. Keinanen-Kiukaanniemi**^197,199^**, Lambertus A. Kiemeney**^97,200,201^**, Diana Kuh**^149^**, Markku Laakso**^126^**, Terho Lehtimäki**^202^**, Douglas F. Levinson**^203^**, Nicholas G. Martin**^32^**, Andres Metspalu**^4,11^**, Andrew D. Morris**^152^**, Markku S. Nieminen**^174^**, Inger Njølstad**^204,205^**, Claes Ohlsson**^81^**, Albertine J. Oldehinkel**^206^**, Willem H. Ouwehand**^5,85,162,163^**, Lyle J. Palmer**^35,36^**, Brenda Penninx**^207^**, Chris Power**^112^**, Michael A. Province**^6^**, Bruce M. Psaty**^55,208,209^**, Lu Qi**^22,23^**, Rainer Rauramaa**^128,210^**, Paul M. Ridker**^68,92^**, Samuli Ripatti**^5,39,66^**, Veikko Salomaa**^125^**, Nilesh J. Samani**^211,212^**, Harold Snieder**^63,80^**, Thorkild I.A. Sørensen**^213^**, Timothy D. Spector**^56^**, Kari Stefansson**^30,214^**, Anke Tönjes**^177,178^**, Jaakko Tuomilehto**^131,215,216,217^**, André G. Uitterlinden**^34,58,59^**, Matti Uusitupa**^218,219^**, Pim van der Harst**^102,137^**, Peter Vollenweider**^183^**, Henri Wallaschofski**^220^**, Nicholas J. Wareham**^10^**, Hugh Watkins**^40^**, H.-Erich Wichmann**^221,222,223^**, James F. Wilson**^91^**, Goncalo R. Abecasis**^13^**, Themistocles L. Assimes**^224^**, Inês Barroso**^5,225^**, Michael Boehnke**^13^**, Ingrid B. Borecki**^6^**, Panos Deloukas**^5^**, Caroline S. Fox**^226^**, Timothy Frayling**^21^**, Leif C. Groop**^227^**, Talin Haritunian**^228^**, Iris M. Heid**^20,221^**, David Hunter**^22,23^**, Robert C. Kaplan**^229^**, Fredrik Karpe**^161,230^**, Miriam Moffatt**^95^**, Karen L. Mohlke**^9^**, Jeffrey R. O'Connell**^231^**, Yudi Pawitan**^2^**, Eric E. Schadt**^232,233^**, David Schlessinger**^234^**, Valgerdur Steinthorsdottir**^30^**, David P. Strachan**^235^**, Unnur Thorsteinsdottir**^30,214^**, Cornelia M. van Duijn**^34,59,236^**, Peter M. Visscher**^25,31^**, Anna Maria Di Blasio**^12^**, Joel N. Hirschhorn**^14,15,16^**, Cecilia M. Lindgren**^3^**, Andrew P. Morris**^3^**, David Meyre**^53,54,237^**, André Scherag**^65^**, Mark I. McCarthy**^3,161,230^**, Elizabeth K. Speliotes**^238,239^***, Kari E. North**^7^***, Ruth J.F. Loos**^10,240,241,242^**, Erik Ingelsson**^2^**

1. Division of Cancer Epidemiology and Genetics, National Cancer Institute, National Institutes of Health, Department of Health and Human Services, Bethesda, Maryland 20892, USA

2. Department of Medical Epidemiology and Biostatistics, Karolinska Institutet, 171 77 Stockholm, Sweden

3. Wellcome Trust Centre for Human Genetics, University of Oxford, Oxford, OX3 7BN, UK

4. Estonian Genome Center, University of Tartu, Tartu 50410, Estonia

5. Wellcome Trust Sanger Institute, Hinxton, Cambridge, CB10 1SA, UK

6. Department of Genetics, Washington University School of Medicine, St Louis, Missouri 63110, USA

7. Department of Epidemiology, School of Public Health, University of North Carolina at Chapel Hill, Chapel Hill, North Carolina 27514, USA

8. Center for Observational Research, Amgen, Thousands Oaks, CA, 91320

9. Department of Genetics, University of North Carolina, Chapel Hill, North Carolina 27599, USA

10. MRC Epidemiology Unit, Institute of Metabolic Science, Addenbrooke's Hospital, Cambridge, CB2 0QQ, UK

11. Institute of Molecular and Cell Biology, University of Tartu, Tartu 51010, Estonia

12. Molecular Biology Department, Istituto Auxologico Italiano, Milano, Italy

13. Department of Biostatistics, Center for Statistical Genetics, University of Michigan, Ann Arbor, Michigan 48109, USA

14. Divisions of Genetics and Endocrinology and Center for Basic and Translational Obesity Research, Children's Hospital, Boston, Massachusetts 02115, USA

15. Metabolism Initiative and Program in Medical and Population Genetics, Broad Institute, Cambridge, Massachusetts 02142, USA

16. Department of Genetics, Harvard Medical School, Boston, Massachusetts 02115, USA

17. Department of Internal Medicine (Cardiovascular), University of Michigan, Ann Arbor, MI 48109, USA

18. Department of Human Genetics, University of Michigan, Ann Arbor, MI 48109, USA

19. Department of Computational Medicine and Bioinformatics, University of Michigan, MI 48109, USA

20. Public Health and Gender Studies, Institute of Epidemiology and Preventive Medicine, Regensburg University Medical Center, Regensburg, Germany

21. Genetics of Complex Traits, Peninsula College of Medicine and Dentistry, University of Exeter, Exeter, EX1 2LU, UK

22. Department of Nutrition, Harvard School of Public Health, Boston, Massachusetts 02115, USA

23. Channing Laboratory, Department of Medicine, Brigham and Women's Hospital and Harvard Medical School, Boston, Massachusetts 02115, USA

24. Department of Biostatistics and Bioinformatics, Emory University, Atlanta, Georgia 30322, USA

25. The Queensland Brain Institute, The University of Queensland, Brisbane, Queensland, Australia

26. Department of Epidemiology, Harvard School of Public Health, Boston, Massachusetts 02115, USA

27. Department of Biostatistics, Harvard School of Public Health, Boston, Massachusetts 02115, USA

28. Department of Biostatistics, University of North Carolina, Chapel Hill, NC 27599, USA

29. Analytic and Translational Genetics Unit, Massachusetts General Hospital, Boston, MA 02114

30. deCODE Genetics, 101 Reykjavik, Iceland

31. University of Queensland Diamantina Institute, University of Queensland, Princess Alexandra Hospital, Brisbane, Queensland 4102, Australia

32. Queensland Institute of Medical Research, Brisbane 4029, Australia

33. Institute of Genetic Epidemiology, Helmholtz Zentrum München - German Research Center for Environmental Health, 85764 Neuherberg, Germany

34. Department of Epidemiology, Erasmus MC, Rotterdam, 3015GE, The Netherlands

35. Genetic Epidemiology and Biostatistics Platform, Ontario Institute for Cancer Research. Toronto, Canada, M5G 1L7

36. Prosserman Centre for Health Research, Samuel Lunenfeld Research Institute, Toronto, Canada, M5G 1X5

37. Centre for Genetic Epidemiology and Biostatistics, University of Western Australia, Crawley, Western Australia 6009, Australia

38. Department of Internal Medicine, VU University Medical Centre, Amsterdam, The Netherlands

39. National Institute for Health and Welfare, Department of Chronic Disease Prevention, Unit of Public Health Genomics, 00014, Helsinki, Finland

40. Cardiovascular Medicine, University of Oxford, Wellcome Trust Centre for Human Genetics, Oxford, OX3 7BN, UK

41. Department of Biological Psychology, VU University Amsterdam, 1081 BT Amsterdam, The Netherlands

42. MRC Human Genetics Unit, MRC Institute for Genetics and Molecular Medicine, Western General Hospital, Edinburgh, EH4 2XU, UK

43. Institute of Medical Biometry and Epidemiology, University of Marburg, 35037 Marburg, Germany

44. Department of Immunology, Genetics and Pathology, Uppsala University, Sweden

45. Uppsala Clinical Research Center, Uppsala university hospital, Sweden

46. Clinical Pharmacology and Barts and The London Genome Centre, William Harvey Research Institute, Barts and The London School of Medicine and Dentistry, Queen Mary University of London, Charterhouse Square, London EC1M 6BQ, UK

47. LURIC Study nonprofit LLC, Freiburg, Germany

48. Mannheim Institute of Public Health, Social and Preventive Medicine, Medical Faculty of Mannheim, University of Heidelberg, Mannheim, Germany

49. Institut für Medizinische Biometrie und Statistik, Universität zu Lübeck, Universitätsklinikum Schleswig-Holstein, Campus Lübeck, 23562 Lübeck, Germany

50. Department of Medical Genetics, University of Lausanne, 1005 Lausanne, Switzerland

51. Swiss Institute of Bioinformatics, 1015 Lausanne, Switzerland

52. Division of Genetic Epidemiology, Department of Medical Genetics, Molecular and Clinical Pharmacology, Innsbruck Medical University, 6020 Innsbruck, Austria

53. University Lille Nord de France, 59000 Lille, France

54. CNRS UMR8199-IBL-Institut Pasteur de Lille, F-59000 Lille, France

55. Cardiovascular Health Research Unit, University of Washington, Seattle, Washington 98101, USA

56. Department of Twin Research and Genetic Epidemiology, King's College London, London, SE1 7EH, UK

57. School of Social and Community Medicine, University of Bristol, UK

58. Department of Internal Medicine, Erasmus MC, Rotterdam, 3015GE, The Netherlands

59. Netherlands Genomics Initiative (NGI)-sponsored Netherlands Consortium for Healthy Aging (NCHA)

60. Department of Medicine I, University Hospital Grosshadern, Ludwig-Maximilians-Universität, Munich, Germany

61. Institute of Medical Informatics, Biometry and Epidemiology, Chair of Epidemiology and Chair of Genetic Epidemiology, Ludwig-Maximilians-Universität, Munich, Germany

62. Department of Biostatistics, Boston University School of Public Health, Boston, Massachusetts 02118, USA

63. Department of Epidemiology, University of Groningen, University Medical Center Groningen, The Netherlands

64. MRC Centre for Causal Analyses in Translational Epidemiology, School of Social and Community Medicine, University of Bristol, Bristol, BS8 2BN, UK

65. Institute for Medical Informatics, Biometry and Epidemiology (IMIBE), University Hospital of Essen, University of Duisburg-Essen, Essen, Germany

66. Institute for Molecular Medicine Finland (FIMM), University of Helsinki, 00014, Helsinki, Finland

67. Universität zu Lübeck, Medizinische Klinik II, 23538 Lübeck, Germany

68. Division of Preventive Medicine, Brigham and Women's Hospital, Boston, Massachusetts 02215, USA

69. Department of Clinical Sciences, Genetic and Molecular Epidemiology Unit, Skåne University Hospital Malmö, Lund University, Malmö, Sweden

70. Department of Public Health & Clinical Medicine, Umeå University,Umeå, Sweden

71. Department of Odontology, Umeå University, Sweden

72. Icelandic Heart Association, Kopavogur, Iceland

73. Department of Medicine, University of Iceland, Reykjavik, Iceland

74. Atherosclerosis Research Unit, Department of Medicine, Solna,Karolinska Institutet, Karolinska University Hospital, 171 76 Stockholm, Sweden

75. Interfaculty Institute for Genetics and Functional Genomics, Ernst-Moritz-Arndt-University Greifswald, 17487 Greifswald, Germany

76. Department of Vascular Medicine, Academic Medical Center, Amsterdam, The Netherlands

77. Heart Failure Research Centre, Department of Clinical and Experimental Cardiology, Academic Medical Center, Amsterdam, the Netherlands

78. Department of Oncology, University of Cambridge, Cambridge, CB1 8RN, UK

79. Department of Endocrinology, University Medical Center Groningen, University of Groningen, P.O. Box 30001, 9700 RB Groningen, The Netherlands

80. LifeLines Cohort Study, University Medical Center Groningen, University of Groningen, The Netherlands

81. Department of Internal Medicine, Institute of Medicine, Sahlgrenska Academy, University of Gothenburg, 413 45 Gothenburg, Sweden

82. Hudson Alpha Institute for Biotechnology, Huntsville, Alabama 35806, USA

83. Department of Cardiology, Division Heart & Lungs, University Medical Center Utrecht, The Netherlands

84. Institute of Biomedicine/Physiology, University of Eastern Finland, Kuopio Campus, Finland

85. NIHR Cambridge Biomedical Research Centre, Cambridge, UK

86. Division of Epidemiology, Multidisciplinary Cardiovascular Research Centre (MCRC), Leeds Institute of Genetics, Health and Therapeutics (LIGHT), University of Leeds, Leeds LS2 9JT, UK

87. PathWest Laboratory of Western Australia, Department of Molecular Genetics, J Block, QEII Medical Centre, Nedlands, Western Australia 6009, Australia

88. Department of Surgery and Pathology, University of Western Australia, Nedlands, Australia, 6009

89. Genome Technology Branch, National Human Genome Research Institute, NIH, Bethesda, MD 20892, USA

90. Dipartimento di Medicina Sperimentale. Università degli Studi Milano-Bicocca, Monza, Italy

91. Centre for Population Health Sciences, University of Edinburgh, Teviot Place, Edinburgh, EH8 9AG, Scotland

92. Harvard Medical School, Boston, Massachusetts 02115, USA

93. British Heart Foundation Glasgow Cardiovascular Research Centre, University of Glasgow, Glasgow, G12 8TA, UK

94. University of Dundee, Ninewells Hospital &Medical School, Dundee, DD1 9SY, UK

95. National Heart and Lung Institute, Imperial College London, London SW3 6LY, UK

96. Division of Cardiovascular Epidemiology, Institute of Environmental Medicine, Karolinska Institutet, Stockholm, Sweden

97. Department of Epidemiology, Biostatistics and HTA, Radboud University Nijmegen Medical Centre, 6500 HB Nijmegen, The Netherlands

98. Istituto di Ricerca Genetica e Biomedicadel del CNR, Monserrato, 09042, Cagliari, Italy

99. Department of Dietetics-Nutrition, Harokopio University, 70 El. Venizelou Str, Athens, Greece

100. Epidemiology and Preventive Medicine Research Center, Department of Clinical and Experimental Medicine, University of Insubria, Varese, Italy

101. Department of Cardiology, Toulouse University School of Medicine, Rangueil Hospital, Toulouse, France

102. Department of Genetics, University Medical Center Groningen, University of Groningen, The Netherlands

103. University of Milan, Department of Health Sciences, Ospedale San Paolo, 20139 Milano, Italy

104. University of Chicago, Chicago, Illinois 60637, USA

105. Northshore University HealthSystem, Evanston, Illinois 60201, USA

106. Research Unit for Molecular Epidemiology, Helmholtz Zentrum München - German Research Center for Environmental Health, Neuherberg, Germany

107. Division of Cardiovascular and Neuronal Remodelling, Multidisciplinary Cardiovascular Research Centre, Leeds Institute of Genetics, Health and Therapeutics, University of Leeds, UK

108. Department of Clinical Sciences/Obstetrics and Gynecology, University of Oulu, 90014 Oulu, Finland

109. Department of Neurology, Boston University School of Medicine, Boston, Massachusetts 02118, USA

110. Department of Psychiatry, Washington University School of Medicine, St Louis, MO 63108, USA

111. Department of Child and Adolescent Psychiatry, University of Duisburg-Essen, 45147 Essen, Germany

112. Centre For Paediatric Epidemiolgy and Biostatistics/MRC Centre of Epidemiology for Child Health, University College of London Institute of Child Health, London, UK

113. Division of Research, Kaiser Permanente Northern California, Oakland, California 94612, USA

114. Core Genotyping Facility, SAIC-Frederick, Inc., NCI-Frederick, Frederick, Maryland 21702, USA

115. Department of Physiology, Institute of Neuroscience and Physiology, Sahlgrenska Academy, University of Gothenburg, 405 30 Gothenburg, Sweden

116. National Institute for Health and Welfare, Department of Chronic Disease Prevention, Population Studies Unit, 20720 Turku, Finland

117. Department of Clinical Physiology, University of Tampere and Tampere University Hospital, 33520 Tampere, Finland;

118. Cardiovascular Research Center and Cardiology Division, Massachusetts General Hospital, Boston, Massachusetts 02114, USA.

119. Center for Human Genetic Research, Massachusetts General Hospital, Boston, Massachusetts 02114, USA.

120. Program in Medical and Population Genetics, Broad Institute of Harvard and Massachusetts Institute of Technology, Cambridge, Massachusetts 02142, USA

121. UKCRC Centre of Excellence for Public Health (NI) Queens University, Belfast

122. Department of Public Health and Primary Care, Institute of Public Health, University of Cambridge, Cambridge CB2 2SR, UK

123. Department of Epidemiology and Public Health, University College London, 1-19 Torrington Place, London WC1E 6BT, UK

124. Department of Internal Medicine II – Cardiology, University of Ulm Medical Center, Ulm, Germany

125. National Institute for Health and Welfare, Department of Chronic Disease Prevention, Chronic Disease Epidemiology and Prevention Unit, 00271, Helsinki, Finland

126. Department of Medicine, University of Eastern Finland, Kuopio Campus and Kuopio University Hospital, 70210 Kuopio, Finland

127. Finnish Institute of Occupational Health, 90220 Oulu, Finland

128. Kuopio Research Institute of Exercise Medicine, Kuopio, Finland

129. Laboratory of Epidemiology, Demography, Biometry, National Institute on Aging, National Institutes of Health, Bethesda, Maryland 20892, USA

130. Department of Medical Sciences, Uppsala University, Akademiska sjukhuset, 751 85 Uppsala, Sweden

131. National Institute for Health and Welfare, Diabetes Prevention Unit, 00271 Helsinki, Finland

132. Human Genetics, Genome Institute of Singapore, Singapore 138672, Singapore

133. Department of Internal Medicine, Istituto Auxologico Italiano, Verbania, Italy

134. Transplantation Laboratory, Haartman Institute, University of Helsinki, 00014, Helsinki, Finland

135. Università Vita-Salute San Raffaele, Chair of Nephrology San Raffaele Scientific Institute, OU Nephrology and Dialysis, 20132 Milan, Italy

136. Synlab Academy, Mannheim, Germany

137. Department of Cardiology, University Medical Center Groningen, University of Groningen, The Netherlands

138. Departments of Biostatistics, University of Washington, Seattle, Washington 98195, USA

139. Genetics Division, GlaxoSmithKline, King of Prussia, Pennsylvania 19406, USA

140. Institute of Human Genetics, University of Bonn, Bonn, Germany

141. Department of Genomics, Life & Brain Center, University of Bonn, Bonn, Germany

142. School of Population Health, The University of Western Australia, Nedlands WA 6009, Australia

143. Department of Respiratory Medicine, Sir Charles Gairdner Hospital, Nedlands, Australia, 6009

144. Busselton Population Medical Research Foundation Inc., Sir Charles Gairdner Hospital, Nedlands, Western Australia 6009, Australia

145. Department of Internal Medicine, University Medical Center Groningen, University of Groningen, Groningen

146. MRC Harwell, Harwell, UK

147. Department of Statistics, University of Oxford, Oxford OX1 3TG, UK

148. Department of Public Health, Section of Epidemiology, Aarhus University, Denmark

149. MRC Unit for Lifelong Health & Ageing, London, UK

150. Department of Clinical Genetics, Erasmus MC, Rotterdam, 3015GE, The Netherlands

151. Centre for Medical Systems Biology & Netherlands Consortium on Healthy Aging, Leiden, the Netherlands

152. Medical Research Institute, University of Dundee, Ninewells Hospital and Medical School. Dundee, DD1 9SY

153. Illumina Inc. Cambridge

154. Institute of Epidemiology II, Helmholtz Zentrum München - German Research Center for Environmental Health, Neuherberg, Germany

155. Munich Heart Alliance, Munich, Germany

156. Faculty of Medicine, University of Split, Croatia

157. National Institute for Health and Welfare, 90101 Oulu, Finland

158. Center for Biomedicine, European Academy Bozen/Bolzano (EURAC), Bolzano/Bozen, 39100, Italy - Affiliated Institute of the University of Lübeck, Lübeck, Germany.

159. Department of Neurology, General Central Hospital, Bolzano, Italy

160. Department of Neurology, University of Lübeck, Lübeck, Germany.

161. Oxford Centre for Diabetes, Endocrinology and Metabolism, University of Oxford, Oxford, OX3 7LJ, UK

162. Department of Haematology, University of Cambridge, Cambridge CB2 0PT, UK

163. NHS Blood and Transplant, Cambridge Centre, Cambridge, CB2 0PT, UK

164. Research Centre of Applied and Preventive Cardiovascular Medicine, University of Turku, 20520 Turku, Finland

165. The Department of Clinical Physiology and Nuclear Medicine, Turku University Hospital, 20520 Turku, Finland

166. MRC Biostatistics Unit, Institute of Public Health, Cambridge, UK

167. Finnish Diabetes Association, Kirjoniementie 15, 33680, Tampere, Finland

168. Pirkanmaa Hospital District, Tampere, Finland

169. South Karelia Central Hospital, 53130 Lappeenranta, Finland

170. Institute for Community Medicine, University Medicine Greifswald, Greifswald, Germany

171. Institute for Clinical Molecular Biology, Christian-Albrechts University, Kiel, Germany

172. Deutsches Zentrum für Herz-Kreislaufforschung e. V. (DZHK), Universität zu Lübeck, 23538 Lübeck, Germany

173. Azienda ospedaliera di Desio e Vimercate, Milano, Italy

174. Division of Cardiology, Cardiovascular Laboratory, Helsinki University Central Hospital, 00029 Helsinki, Finland

175. University of Eastern Finland and Kuopio University Hospital, 70210 Kuopio, Finland

176. Klinik und Poliklinik für Innere Medizin II, Universitätklinikum Regensburg, 93053 Regensburg, Germany

177. Department of Medicine, University of Leipzig, 04103 Leipzig, Germany

178. University of Leipzig, IFB Adiposity Diseases, Leipzig, Germany

179. INSERM UMR_S 937, ICAN Institute, Pierre et Marie Curie Medical School, Paris 75013, France

180. Dipartimento di Scienze Farmacologiche e Biomolecolari, Università di Milano, Centro Cardiologico Monzino, IRCCS, Milan, Italy

181. Department of Human Genetics, Radboud University Nijmegen Medical Centre, PO Box 9101, 6500 HB Nijmegen, The Netherlands

182. Department of Medicine, University of Turku and Turku University Hospital, 20520 Turku, Finland

183. Department of Internal Medicine, Centre Hospitalier Universitaire Vaudois (CHUV) University Hospital, 1011 Lausanne, Switzerland

184. Cardiology Group, Frankfurt-Sachsenhausen, Germany

185. Institut Pasteur de Lille, INSERM U744, Université Lille Nord de France, F-59000 Lille, France

186. Division of Endocrinology and Diabetes, Department of Medicine, University Hospital, Ulm, Germany

187. Human Genetics Center and Institute of Molecular Medicine, University of Texas Health Science Center, Houston, Texas 77030, USA

188. Fondazione Filarete, Milano, Italy

189. Department of General Practice and Primary health Care, University of Helsinki, Helsinki, Finland

190. National Institute for Health and Welfare, 00271 Helsinki, Finland

191. Helsinki University Central Hospital, Unit of General Practice, 00280 Helsinki, Finland

192. Department of Nutrition, Harvard School of Public Health, Boston, MA

193. Department of Genomics of Common Disease, School of Public Health, Imperial College London, W12 0NN, London, UK

194. HUNT Research Centre, Department of Public Health and General Practice, Norwegian University of Science and Technology, 7600 Levanger, Norway

195. Hannover Unified Biobank, Hannover Medical School, 30625 Hannover, Germany

196. Department of Epidemiology and Biostatistics, School of Public Health, Faculty of Medicine, Imperial College London, London, W2 1PG, UK

197. Institute of Health Sciences, University of Oulu, 90014 Oulu, Finland

198. Biocenter Oulu, University of Oulu, 90014 Oulu, Finland

199. Unit of General Practice, Oulu University Hospital, Oulu, Finland

200. Department of Urology, Radboud University Nijmegen Medical Centre, 6500 HB Nijmegen, The Netherlands

201. Comprehensive Cancer Center East, 6501 BG Nijmegen, The Netherlands

202. Department of Clinical Chemistry, Fimlab Laboratories, University of Tampere and Tampere University Hospital, 33520 Tampere, Finland

203. Stanford University School of Medicine, Stanford, California 93405, USA

204. Department of Clinical Medicine, Faculty of Health Sciences, University of Tromsø, Tromsø, Norway

205. Department of Community Medicine, Faculty of Health Sciences, University of Tromsø, Tromsø, Norway

206. Interdisciplinary Center Psychopathology and Emotion Regulation, University of Groningen, University Medical Center Groningen, The Netherlands

207. Department of Psychiatry, University Medical Centre Groningen, 9713 GZ Groningen, The Netherlands

208. Departments of Epidemiology, Medicine and Health Services, University of Washington, Seattle, Washington 98195, USA

209. Group Health Research Institute, Group Health, Seattle, Washington 98101, USA

210. Department of Clinical Physiology and Nuclear Medicine, Kuopio University Hospital, Kuopio, Finland

211. Department of Cardiovascular Sciences, University of Leicester, Glenfield Hospital, Leicester, LE3 9QP, UK

212. Leicester NIHR Biomedical Research Unit in Cardiovascular Disease, Glenfield Hospital, Leicester, LE3 9QP, UK

213. Institute of Preventive Medicine, Bispebjerg University Hospital, Copenhagen, and Novo Nordisk Foundation Center for Basic Metabolic Research, University of Copenhagen, Denmark

214. Faculty of Medicine, University of Iceland, 101 Reykjavík, Iceland

215. Red RECAVA Grupo RD06/0014/0015, Hospital Universitario La Paz, 28046 Madrid, Spain

216. Centre for Vascular Prevention, Danube-University Krems, 3500 Krems, Austria

217. South Ostrobothnia Central Hospital, 60220 Seinajoki, Finland

218. Institute of Public Health and Clinical Nutrition, University of Eastern Finland, Finland

219. Research Unit, Kuopio University Hospital, Kuopio, Finland

220. Institute of Clinical Chemistry and Laboratory Medicine, University Medicine Greifswald, 17475 Greifswald, Germany

221. Institute of Epidemiology I, Helmholtz Zentrum München - German Research Center for Environmental Health, Neuherberg, Germany

222. Institute of Medical Informatics, Biometry and Epidemiology, Chair of Epidemiology, Ludwig-Maximilians-Universität, Munich, Germany

223. Klinikum Grosshadern, Munich, Germany

224. Department of Medicine, Stanford University School of Medicine, Stanford, California 94305, USA

225. University of Cambridge Metabolic Research Labs, Institute of Metabolic Science Addenbrooke's Hospital, CB2 OQQ, Cambridge, UK

226. Division of Intramural Research, National Heart, Lung and Blood Institute, Framingham Heart Study, Framingham, Massachusetts 01702, USA

227. Lund University Diabetes Centre, Department of Clinical Sciences, Lund University, 20502 Malmö, Sweden

228. Medical Genetics Institute, Cedars-Sinai Medical Center, Los Angeles, California 90048, USA

229. Department of Epidemiology and Population Health, Albert Einstein College of Medicine, Bronx, New York 10461, USA

230. Oxford National Institute for Health Research Biomedical Research Centre, Churchill Hospital, Old Road Headington, Oxford, OX3 7LJ, UK

231. Department of Medicine, University of Maryland School of Medicine, Baltimore, Maryland 21201, USA

232. Department of Genetics and Genomic Sciences, Mount Sinai School of Medicine, One Gustave L. Levy Place, Box 1498, New York, NY 10029-6574 USA

233. Institute of Genomics and Multiscale Biology, Mount Sinai School of Medicine, One Gustave L. Levy Place, Box 1498, New York, NY 10029-6574 USA

234. Laboratory of Genetics, National Institute on Aging, Baltimore, Maryland 21224, USA

235. Division of Population Health Sciences and Education, St George's, University of London, London, SW17 0RE, UK

236. Center of Medical Systems Biology, Leiden University Medical Center, 2333 ZC Leiden, the Netherlands

237. Department of Clinical Epidemiology and Biostatistics, McMasterUniversity, Hamilton, Ontario L8S 4L8, Canada

238. Center for Computational Medicine and Bioinformatics, University of Michigan, Ann Arbor, Michigan, USA

239. Department of Internal Medicine, Division of Gastroenterology, University of Michigan, Ann Arbor, Michigan, USA

240. The Charles Bronfman Institute of Personalized Medicine, Mount Sinai School of Medicine, New York, NY 10029, USA

241. Child Health and Development Institute, Mount Sinai School of Medicine, New York, NY 10029, USA

242. Department of Preventive Medicine, Mount Sinai School of Medicine, New York, NY 10029, USA

**
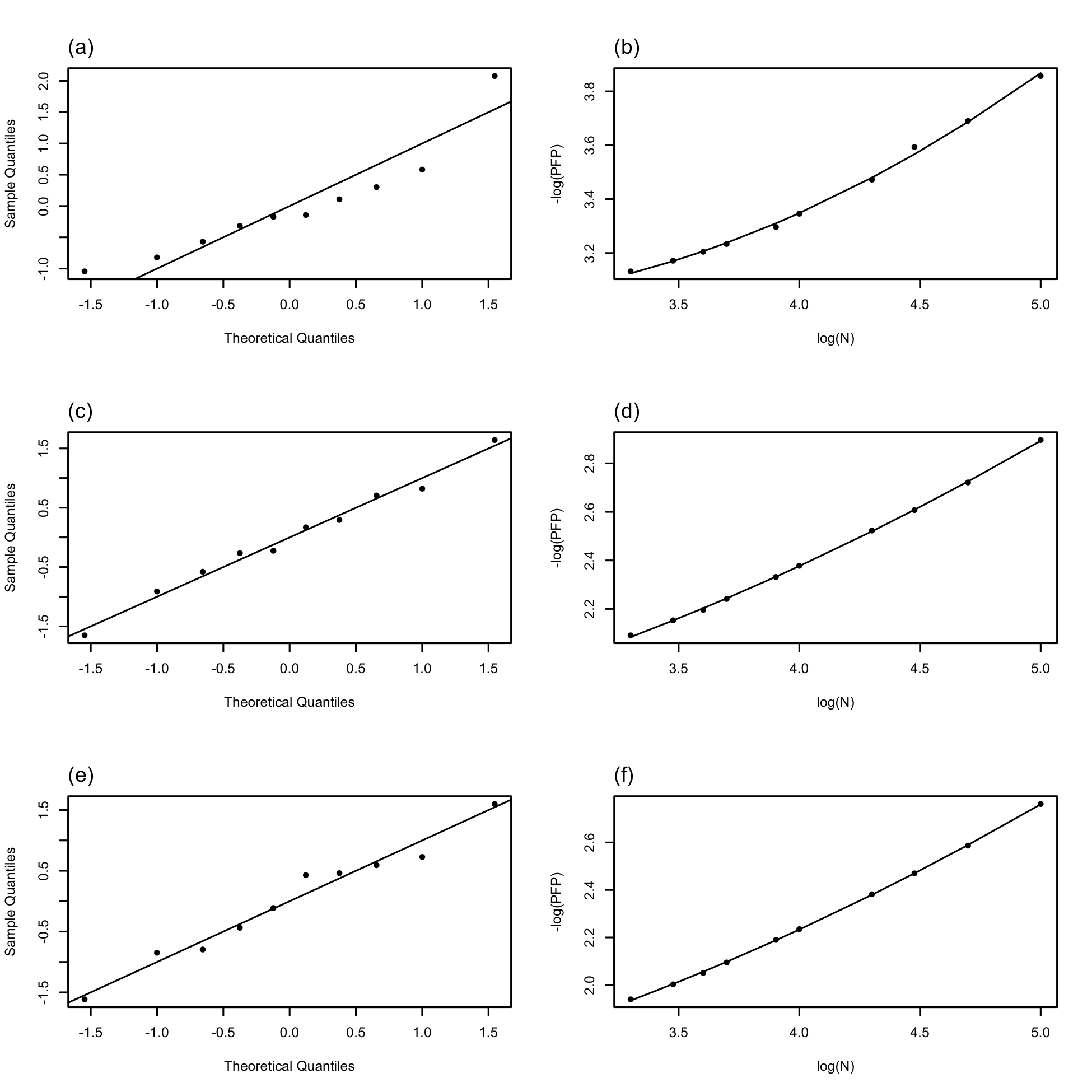
**

**Figure S1:** Standardised residual QQ-plots to assess normality are provided in the left panel for the regression models with R=2 (a), R=15 (c) and R=20 (e). The right panel displays corresponding plots of the estimated values (from simulations) of –log_10_(PFP) against log_10_(N) with the fitted –log_10_(PFP) regression line for R=2 (b), R=15 (d) and R=20 (f).

Table S 1: Type I error estimates using a Bayesian threshold log_10_θ, determined from π_0_ = 0.999 and varying R. Estimates are based on 5 million independent SNPs from equal-sized case-control samples, each of size N. Estimates at N=15,000 are the result of a regression at each R value of the PFP estimates against a quadratic of log_10_N.

| N\R (log_10_θ) | 1 (3.000) | 2 (2.699) | 4 (2.398) | 5 (2.301) | 10 (2.000) | 12 (1.920) | 15 (1.823) | 20 (1.699) |
| --- | --- | --- | --- | --- | --- | --- | --- | --- |
| 2,000 | 2.38 E-05 | 5.28 E-05 | 1.14 E-04 | 1.49 E-04 | 3.31 E-04 | 4.01 E-04 | 5.18 E-04 | 7.30 E-04 |
| 3,000 | 2.22 E-05 | 5.04 E-05 | 1.09 E-04 | 1.44 E-04 | 3.08 E-04 | 3.75 E-04 | 4.87 E-04 | 6.68 E-04 |
| 4,000 | 1.80 E-05 | 4.06 E-05 | 9.12 E-05 | 1.16 E-04 | 2.74 E-04 | 3.41 E-04 | 4.45 E-04 | 6.18 E-04 |
| 5,000 | 1.94 E-05 | 4.36 E-05 | 1.01 E-04 | 1.27 E-04 | 2.74 E-04 | 3.36 E-04 | 4.27 E-04 | 5.79 E-04 |
| 8,000 | 1.78 E-05 | 4.18 E-05 | 8.60 E-05 | 1.13 E-04 | 2.31 E-04 | 2.83 E-04 | 3.64 E-04 | 4.98 E-04 |
| 10,000 | 1.82 E-05 | 3.58 E-05 | 7.58 E-05 | 1.00 E-04 | 2.14 E-04 | 2.52 E-04 | 3.23 E-04 | 4.45 E-04 |
| 15,000^*^ | 1.28E-05 | 2.81E-05 | 6.03E-05 | 7.92E-05 | 1.77E-04 | 2.15E-04 | 2.73E-04 | 3.74E-04 |
| 20,000 | 1.20 E-05 | 2.60 E-05 | 5.56 E-05 | 7.26 E-05 | 1.60 E-04 | 1.94 E-04 | 2.41 E-04 | 3.34 E-04 |
| 30,000 | 8.60 E-06 | 1.92 E-05 | 4.34 E-05 | 6.08 E-05 | 1.21 E-04 | 1.46 E-04 | 1.86 E-04 | 2.53 E-04 |
| 50,000 | 8.40 E-06 | 1.66 E-05 | 3.36 E-05 | 4.28 E-05 | 9.52 E-05 | 1.15 E-04 | 1.47 E-04 | 2.03 E-04 |
| 100,000 | 6.40 E-06 | 1.24 E-05 | 2.56 E-05 | 3.42 E-05 | 6.66 E-05 | 7.96 E-05 | 1.00 E-04 | 1.3E-04 |

Table S 2: Regression coefficient estimates from model

together with their standard errors σ_i_. All coefficient estimates have p-value < 0.01.

| \| π_0_=0.99 \| \| \| \| \| --- \| --- \| --- \| --- \| \| R \| β__ (σ_0_) \| β_1_ (σ_1_) \| β_2_ (σ_2_) \| \| 1 \| 4.211 (0.334) \| -0.650 (0.163) \| 0.129 (0.0197) \| \| 2 \| 3.619 (0.274) \| -0.537 (0.134) \| 0.117 (0.0162) \| \| 4 \| 2.827 (0.221) \| -0.318 (0.108) \| 0.0914 (0.0130) \| \| 5 \| 2.724 (0.148) \| -0.327 (0.072) \| 0.093 (0.00877) \| \| 10 \| 1.975 (0.0930) \| -0.142 (0.0455) \| 0.0729 (0.00549) \| \| 12 \| 1.583 (0.00726) \| 0 \| 0.0565 (0.000421) \| \| 15 \| 1.458 (0.00538) \| 0 \| 0.0573 (0.000312) \| \| 20 \| 1.295 (0.00403) \| 0 \| 0.0586 (0.000234) \| | \| π_0_=0.999 \| \| \| \| \| --- \| --- \| --- \| --- \| \| R \| β__ (σ_0_) \| β_1_ (σ_1_) \| β_2_ (σ_2_) \| \| 1 \| 3.433 (0.121) \| 0.349 (0.0297) \| 0 \| \| 2 \| 3.734 (.0484) \| 0 \| 0.0468 (0.00281) \| \| 4 \| 3.368 (0.0421) \| 0 \| 0.0487 (0.00244) \| \| 5 \| 3.267 (0.0442) \| 0 \| 0.0478 (0.00257) \| \| 10 \| 4.192 (0.332) \| -0.639 (0.162) \| 0.127 (0.0196) \| \| 12 \| 4.100 (0.322) \| -0.640 (0.157) \| 0.128 (0.0190) \| \| 15 \| 3.902 (0.276) \| -0.603 (0.135) \| 0.125 (0.0163) \| \| 20 \| 3.602 (0.266) \| -0.526 (0.130) \| 0.115 (0.0157) \| |
| --- | --- | --- | --- | --- | --- | --- | --- | --- | --- | --- | --- | --- | --- | --- | --- | --- | --- | --- | --- | --- | --- | --- | --- | --- | --- | --- | --- | --- | --- | --- | --- | --- | --- | --- | --- | --- | --- | --- | --- | --- | --- | --- | --- | --- | --- | --- | --- | --- | --- | --- | --- | --- | --- | --- | --- | --- | --- | --- | --- | --- | --- | --- | --- | --- | --- | --- | --- | --- | --- | --- | --- | --- | --- | --- | --- | --- | --- | --- | --- | --- | --- |

Table S 3: Overlap power results coinciding with Figure 3, where N_i_ denotes the sample size for each of the cases and controls in study *i*, OR_i_ denotes the OR of the causal variant in study *i*, which has the specified MAF.

| \| (a) N_1_ = 5,000; N_2_ = 10,000;  OR_1_ = 1.1; OR_2_ = 1.2  MAF = 0.1 \| \| \| \| \| --- \| --- \| --- \| --- \| \|  \| Power \| \| \| \| R \| Lower α \| ABF \| Upper α \| \| 1 \| 0.052 \| 0.065 \| 0.059 \| \| 2 \| 0.075 \| 0.096 \| 0.086 \| \| 4 \| 0.110 \| 0.141 \| 0.127 \| \| 5 \| 0.122 \| 0.159 \| 0.140 \| \| 10 \| 0.178 \| 0.226 \| 0.202 \| \| 12 \| 0.195 \| 0.248 \| 0.221 \| \| 15 \| 0.216 \| 0.276 \| 0.246 \| \| 20 \| 0.248 \| 0.316 \| 0.283 \| | \| (b) N_1_ = 10,000; N_2_ = 20,000;  OR_1_ = 1.1; OR_2_ = 1.1  MAF = 0.1 \| \| \| \| \| --- \| --- \| --- \| --- \| \|  \| Power \| \| \| \| R \| Lower α \| ABF \| Upper α \| \| 1 \| 0.132 \| 0.165 \| 0.154 \| \| 2 \| 0.188 \| 0.232 \| 0.213 \| \| 4 \| 0.257 \| 0.315 \| 0.290 \| \| 5 \| 0.282 \| 0.345 \| 0.316 \| \| 10 \| 0.373 \| 0.448 \| 0.420 \| \| 12 \| 0.398 \| 0.478 \| 0.447 \| \| 15 \| 0.434 \| 0.515 \| 0.481 \| \| 20 \| 0.481 \| 0.563 \| 0.528 \| |
| --- | --- | --- | --- | --- | --- | --- | --- | --- | --- | --- | --- | --- | --- | --- | --- | --- | --- | --- | --- | --- | --- | --- | --- | --- | --- | --- | --- | --- | --- | --- | --- | --- | --- | --- | --- | --- | --- | --- | --- | --- | --- | --- | --- | --- | --- | --- | --- | --- | --- | --- | --- | --- | --- | --- | --- | --- | --- | --- | --- | --- | --- | --- | --- | --- | --- | --- | --- | --- | --- | --- | --- | --- | --- | --- | --- | --- | --- | --- | --- | --- | --- | --- | --- | --- | --- | --- | --- | --- | --- |
| \| (c) N_1_ = 5,000; N_2_ = 20,000;  OR_1_ = 1.2; OR_2_ = 1.2  MAF = 0.1 \| \| \| \| \| --- \| --- \| --- \| --- \| \|  \| Power \| \| \| \| R \| Lower α \| ABF \| Upper α \| \| 1 \| 0.608 \| 0.679 \| 0.662 \| \| 2 \| 0.680 \| 0.750 \| 0.729 \| \| 4 \| 0.747 \| 0.815 \| 0.799 \| \| 5 \| 0.767 \| 0.835 \| 0.815 \| \| 10 \| 0.828 \| 0.889 \| 0.874 \| \| 12 \| 0.843 \| 0.902 \| 0.886 \| \| 15 \| 0.861 \| 0.916 \| 0.902 \| \| 20 \| 0.883 \| 0.932 \| 0.920 \| | \| (d) N_1_ = 10,000; N_2_ = 30,000;  OR_1_ = 1.1; OR_2_ = 1.1  MAF = 0.2 \| \| \| \| \| --- \| --- \| --- \| --- \| \|  \| Power \| \| \| \| R \| Lower α \| ABF \| Upper α \| \| 1 \| 0.523 \| 0.583 \| 0.583 \| \| 2 \| 0.596 \| 0.659 \| 0.655 \| \| 4 \| 0.674 \| 0.732 \| 0.728 \| \| 5 \| 0.700 \| 0.755 \| 0.748 \| \| 10 \| 0.774 \| 0.821 \| 0.818 \| \| 12 \| 0.789 \| 0.836 \| 0.834 \| \| 15 \| 0.812 \| 0.855 \| 0.852 \| \| 20 \| 0.837 \| 0.877 \| 0.875 \| |

Table S 4: Overlap power results based on R=20 and π_0_=0.99 for the ABF threshold and coinciding p-value threshold (from Table 2) , where N_i_ denotes the sample size for each of the cases and controls in study *i*, OR_i_ denotes the OR of the causal variant in study *i*, which has MAF 0.1.

| MAF 0.1 | | Power | | |
| --- | --- | --- | --- | --- |
| (N_1_, N_2_) | (OR_1_, OR_2_) | Lower α | ABF | Upper α |
| 5,000  10,000 | 1.1, 1.1 | 0.143 | 0.194 | 0.175 |
|  | 1.2, 1.2 | 0.901 | 0.931 | 0.919 |
|  | 1.1, 1.2 | 0.248 | 0.316 | 0.283 |
|  | 1.2, 1.1 | 0.518 | 0.571 | 0.566 |
| 5,000  15,000 | 1.1, 1.1 | 0.180 | 0.257 | 0.236 |
|  | 1.2, 1.2 | 0.891 | 0.932 | 0.920 |
|  | 1.1, 1.2 | 0.230 | 0.316 | 0.284 |
|  | 1.2, 1.1 | 0.700 | 0.757 | 0.764 |
| 5,000  20,000 | 1.1, 1.1 | 0.196 | 0.290 | 0.265 |
|  | 1.2, 1.2 | 0.883 | 0.932 | 0.920 |
|  | 1.1, 1.2 | 0.217 | 0.316 | 0.284 |
|  | 1.2, 1.1 | 0.798 | 0.856 | 0.860 |
| 10,000  20,000 | 1.1, 1.1 | 0.481 | 0.563 | 0.528 |
|  | 1.2, 1.2 | 0.998 | 0.999 | 0.999 |
|  | 1.1, 1.2 | 0.533 | 0.613 | 0.574 |
|  | 1.2, 1.1 | 0.902 | 0.918 | 0.919 |
| 10,000  30,000 | 1.1, 1.1  1.2, 1.1 | 0.499  0.983 | 0.606  0.987 | 0.568  0.989 |

Table S 5: Overlap power results for the scenario where the causal variant has MAF 0.2 and a larger effect in the smaller sample: OR_1_ = 1.2 and OR_2_ = 1.1. The sample size of each of the cases and controls in study *i*, is denoted by N_i._

| \| (a) N_1_ = 5,000; N_2_ = 10,000; \| \| \| \| \| --- \| --- \| --- \| --- \| \|  \| Power \| \| \| \| R \| Lower α \| ABF \| Upper α \| \| 1 \| 0.556 \| 0.560 \| 0.582 \| \| 2 \| 0.634 \| 0.642 \| 0.661 \| \| 4 \| 0.714 \| 0.720 \| 0.744 \| \| 5 \| 0.736 \| 0.745 \| 0.764 \| \| 10 \| 0.811 \| 0.815 \| 0.835 \| \| 12 \| 0.828 \| 0.832 \| 0.851 \| \| 15 \| 0.847 \| 0.852 \| 0.870 \| \| 20 \| 0.871 \| 0.874 \| 0.893 \| | \| (b) N_1_ = 5,000; N_2_ = 15,000; \| \| \| \| \| --- \| --- \| --- \| --- \| \|  \| Power \| \| \| \| R \| Lower α \| ABF \| Upper α \| \| 1 \| 0.806 \| 0.817 \| 0.839 \| \| 2 \| 0.860 \| 0.870 \| 0.886 \| \| 4 \| 0.905 \| 0.912 \| 0.927 \| \| 5 \| 0.915 \| 0.922 \| 0.936 \| \| 10 \| 0.948 \| 0.952 \| 0.963 \| \| 12 \| 0.955 \| 0.958 \| 0.968 \| \| 15 \| 0.962 \| 0.965 \| 0.974 \| \| 20 \| 0.970 \| 0.972 \| 0.981 \| |
| --- | --- | --- | --- | --- | --- | --- | --- | --- | --- | --- | --- | --- | --- | --- | --- | --- | --- | --- | --- | --- | --- | --- | --- | --- | --- | --- | --- | --- | --- | --- | --- | --- | --- | --- | --- | --- | --- | --- | --- | --- | --- | --- | --- | --- | --- | --- | --- | --- | --- | --- | --- | --- | --- | --- | --- | --- | --- | --- | --- | --- | --- | --- | --- | --- | --- | --- | --- | --- | --- | --- | --- | --- | --- | --- | --- | --- | --- | --- | --- | --- | --- | --- | --- | --- | --- | --- | --- | --- | --- |
| \| (c) N_1_ = 5,000; N_2_ = 20,000; \| \| \| \| \| --- \| --- \| --- \| --- \| \|  \| Power \| \| \| \| R \| Lower α \| ABF \| Upper α \| \| 1 \| 0.906 \| 0.921 \| 0.930 \| \| 2 \| 0.937 \| 0.948 \| 0.955 \| \| 4 \| 0.960 \| 0.969 \| 0.974 \| \| 5 \| 0.966 \| 0.974 \| 0.978 \| \| 10 \| 0.980 \| 0.986 \| 0.988 \| \| 12 \| 0.983 \| 0.988 \| 0.990 \| \| 15 \| 0.986 \| 0.990 \| 0.992 \| \| 20 \| 0.990 \| 0.993 \| 0.995 \| | \| (d) N_1_ = 10,000; N_2_ = 20,000; \| \| \| \| \| --- \| --- \| --- \| --- \| \|  \| Power \| \| \| \| R \| Lower α \| ABF \| Upper α \| \| 1 \| 0.960 \| 0.961 \| 0.967 \| \| 2 \| 0.974 \| 0.974 \| 0.979 \| \| 4 \| 0.984 \| 0.985 \| 0.987 \| \| 5 \| 0.986 \| 0.987 \| 0.989 \| \| 10 \| 0.992 \| 0.993 \| 0.994 \| \| 12 \| 0.994 \| 0.994 \| 0.995 \| \| 15 \| 0.995 \| 0.995 \| 0.996 \| \| 20 \| 0.996 \| 0.996 \| 0.997 \| |

Table S 6: Probabilities conditional on overlap SNPs detected by ABF or upper α, α_U_ for various values of R and π_0_=0.99 where probabilities conditional on this set of SNPs are denoted *P^C^.*  The probability of detection by either approach is denoted __while __ is the probability of detection by ABF given that the SNP was identified by either ABF or α_U_ and __ is the conditional probability of detection by ABF and not α_U_. Analogous notation is defined for conditional probabilities related to detection by α_U_.

|  | __ | __ | __ | __ | __ |  |
| --- | --- | --- | --- | --- | --- | --- |
| N_1_ = 10,000;  N_2_ = 20,000;  OR_1_ = 1.1;  OR_2_ = 1.1;  MAF = 0.1 | 1 | 0.166 | 0.994 | 0.925 | 0.075 | 0.0060 |
|  | 2 | 0.232 | 0.997 | 0.916 | 0.084 | 0.0026 |
|  | 4 | 0.315 | 0.998 | 0.920 | 0.080 | 0.0024 |
|  | 5 | 0.345 | 0.999 | 0.915 | 0.085 | 0.0010 |
|  | 10 | 0.450 | 0.997 | 0.933 | 0.067 | 0.0033 |
|  | 12 | 0.479 | 0.998 | 0.934 | 0.066 | 0.0023 |
|  | 15 | 0.515 | 0.998 | 0.934 | 0.066 | 0.0016 |
|  | 20 | 0.564 | 0.998 | 0.936 | 0.064 | 0.0021 |
| N_1_ = 5,000;  N_2_ = 10,000;  OR_1_ = 1.2;  OR_2_ = 1.1;  MAF = 0.2 | 1 | 0.583 | 0.961 | 0.999 | 0.0012 | 0.039 |
|  | 2 | 0.662 | 0.970 | 0.999 | 0.0017 | 0.030 |
|  | 4 | 0.744 | 0.969 | 1.000 | 0.0004 | 0.031 |
|  | 5 | 0.764 | 0.975 | 0.999 | 0.0009 | 0.025 |
|  | 10 | 0.835 | 0.976 | 1.000 | 0.0003 | 0.024 |
|  | 12 | 0.851 | 0.977 | 1.000 | 0.0003 | 0.023 |
|  | 15 | 0.870 | 0.979 | 1.000 | 0.0002 | 0.021 |
|  | 20 | 0.893 | 0.979 | 1.000 | 0.0001 | 0.021 |
| N_1_ = 5,000;  N_2_ = 15,000;  OR_1_ = 1.2;  OR_2_ = 1.1;  MAF = 0.2 | 1 | 0.840 | 0.973 | 0.999 | 0.0007 | 0.027 |
|  | 2 | 0.887 | 0.980 | 0.999 | 0.0011 | 0.020 |
|  | 4 | 0.928 | 0.983 | 1.000 | 0.0003 | 0.017 |
|  | 5 | 0.937 | 0.985 | 0.999 | 0.0007 | 0.015 |
|  | 10 | 0.963 | 0.988 | 1.000 | 0.0002 | 0.012 |
|  | 12 | 0.969 | 0.989 | 1.000 | 0.0002 | 0.011 |
|  | 15 | 0.974 | 0.991 | 1.000 | 0.0002 | 0.009 |
|  | 20 | 0.981 | 0.992 | 1.000 | 0.0001 | 0.008 |

Table S 7: Number of overlap variants identified by the ABF (log_10_θ threshold), according to various R values with π_0_=0.999 and the corresponding lower and upper p-value thresholds, based on 30,000 and 8,000 cases, respectively. The McNemar p-value follows the counts of detected overlap variants.

| ABF | | | Lower α | | Upper α | |
| --- | --- | --- | --- | --- | --- | --- |
| R | log_10_θ  threshold | # detected  (McNemar  mid-p-value) | α_L_  threshold  (N=30,000) | # detected  (McNemar  mid-p-value) | α_U_  threshold  (N=8,000) | # detected  (McNemar  mid-p-value) |
| 1 | 3.000 | 1  (8.21 E-04) | 8.60 E-06 | 1  (5.66 E-06) | 1.80 E-05 | 1  (1.16E-05) |
| 2 | 2.699 | 1  ( 6.30 E-02) | 1.90 E-05 | 1  (3.39 E-06) | 4.20 E-05 | 1  (1.18E-03) |
| 4 | 2.398 | 2  (9.50 E-01) | 4.30 E-05 | 1  (1.25 E-03) | 8.60 E-05 | 2  (1.79E-02) |
| 5 | 2.301 | 2  (5.98 E-01) | 6.10 E-05 | 2  (5.00 E-03) | 1.10 E-04 | 2  (1.58E-01) |
| 10 | 2.000 | 2  (1.90 E-03) | 1.20 E-04 | 2  (1.53 E-01) | 2.30 E-04 | 2  (8.24E-01) |
| 12 | 1.920 | 2  (7.83 E-05) | 1.50 E-04 | 2  (3.30 E-01) | 2.80 E-04 | 2  (6.22E-01) |
| 15 | 1.823 | 2  (4.46 E-07) | 1.90 E-04 | 2  (8.87 E-01) | 3.60 E-04 | 2  (2.42E-01) |
| 20 | 1.699 | 2  (6.42 E-10) | 2.50 E-04 | 2  (6.98 E-01) | 5.00 E-04 | 2  (4.77E-02) |
